# Passive Infrared Sensor-Based Occupancy Monitoring in Smart Buildings: A Review of Methodologies and Machine Learning Approaches

**DOI:** 10.3390/s24051533

**Published:** 2024-02-27

**Authors:** Azad Shokrollahi, Jan A. Persson, Reza Malekian, Arezoo Sarkheyli-Hägele, Fredrik Karlsson

**Affiliations:** 1 Internet of Things and People Research Center, Department of Computer Science and Media Technology, Malmö University, 211 19 Malmö, Sweden; jan.a.persson@mau.se (J.A.P.); reza.malekian@mau.se (R.M.); arezoo.sarkheyli-haegele@mau.se (A.S.-H.); 2 Sony Network Communications, 223 62 Lund, Sweden; fredrik.karlsson@sony.com

**Keywords:** passive infrared sensors (PIR), smart buildings, IoT (internet of things), occupancy information, people counting, activity detection, machine learning

## Abstract

Buildings are rapidly becoming more digitized, largely due to developments in the internet of things (IoT). This provides both opportunities and challenges. One of the central challenges in the process of digitizing buildings is the ability to monitor these buildings’ status effectively. This monitoring is essential for services that rely on information about the presence and activities of individuals within different areas of these buildings. Occupancy information (including people counting, occupancy detection, location tracking, and activity detection) plays a vital role in the management of smart buildings. In this article, we primarily focus on the use of passive infrared (PIR) sensors for gathering occupancy information. PIR sensors are among the most widely used sensors for this purpose due to their consideration of privacy concerns, cost-effectiveness, and low processing complexity compared to other sensors. Despite numerous literature reviews in the field of occupancy information, there is currently no literature review dedicated to occupancy information derived specifically from PIR sensors. Therefore, this review analyzes articles that specifically explore the application of PIR sensors for obtaining occupancy information. It provides a comprehensive literature review of PIR sensor technology from 2015 to 2023, focusing on applications in people counting, activity detection, and localization (tracking and location). It consolidates findings from articles that have explored and enhanced the capabilities of PIR sensors in these interconnected domains. This review thoroughly examines the application of various techniques, machine learning algorithms, and configurations for PIR sensors in indoor building environments, emphasizing not only the data processing aspects but also their advantages, limitations, and efficacy in producing accurate occupancy information. These developments are crucial for improving building management systems in terms of energy efficiency, security, and user comfort, among other operational aspects. The article seeks to offer a thorough analysis of the present state and potential future advancements of PIR sensor technology in efficiently monitoring and understanding occupancy information by classifying and analyzing improvements in these domains.

## 1. Introduction

In the context of the internet of things (IoT), smart buildings are defined as structures utilizing interconnected devices and sensors to collect, share, and analyze data. This data-centric approach enhances operational efficiency, energy management, and the overall user experience by optimizing various aspects of building management [1]. Such an approach is central to the systematic improvement of energy use and operational processes in modern facilities [2,3], extending beyond architectural design to encompass comprehensive building management and user comfort [4]. The real-time monitoring [5] and management of building systems [6], such as lighting, heating, ventilation, and security, are made possible by the IoT, which ranges from sophisticated cameras to different types of sensors [7,8,9]. The capacity to interpret this data using cutting-edge algorithms and artificial intelligence (AI) techniques enables smart buildings to “learn” and adapt to the preferences and behaviors of their occupants. This adaptability ensures that smart buildings’ environment is constantly ideal, improving user comfort, energy efficiency, and overall experience [10,11,12,13]. AI integration also makes predictive maintenance easier, allowing for the early detection and resolution of possible problems to ensure the longevity and effectiveness of building systems [14,15,16,17]. Essentially, smart buildings offer a sustainable, efficient, and user-centric environment that combines the capabilities of the IoT and AI [18,19]. In smart buildings, occupancy information is critical for shaping energy consumption, optimizing HVAC and lighting systems for energy efficiency, improving occupant comfort through personalized environments, strengthening security by detecting unexpected occupancy in real time, and making design adjustments based on space utilization patterns. This information, which depicts the presence, movement, and activities of humans within specified places like rooms or buildings, is used for resource efficiency, user comfort, and successful building management [20]. The applications of occupancy information include smart buildings, intelligent transportation systems, building management and automation, healthcare and monitoring, and energy-efficient infrastructure [21,22]. While these areas are vast, this article particularly emphasizes the role of smart buildings, concentrating on how such data can revolutionize building operations, comfort, and so on.

**Figure 1:**
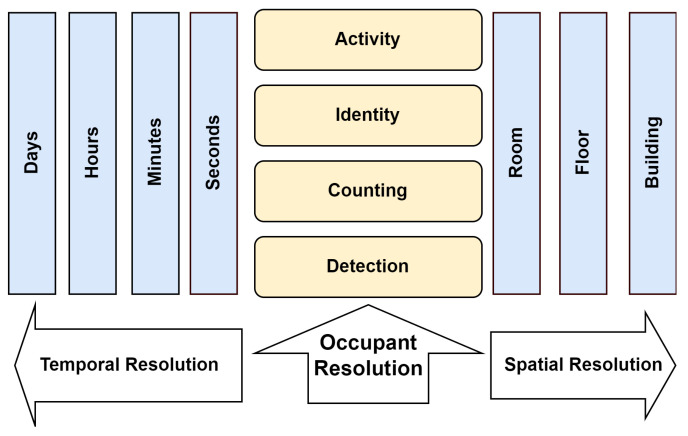
Levels of occupancy information [20].

The levels of occupancy information mentioned by Melfi et al. [23] include presence, counting, location, tracking, and identity. These levels are presence (occupancy), counting, identity, and activity [20], as you can see in Figure 1.

**Figure 2:**
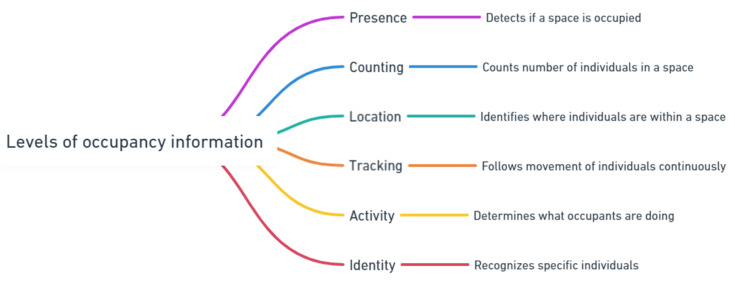
Updated levels of occupancy information.

The authors introduced a three-dimensional concept of presence. These dimensions include occupant resolution, spatial resolution, and temporal resolution. When it comes to determining the presence of people, higher resolution is directly related to improved accuracy and a more comprehensive understanding of both occupancy and the activities of the occupants. This multi-dimensional approach provides a comprehensive framework to analyze and interpret occupancy data, allowing for a more nuanced understanding of how spaces are utilized over time and across different locations. Different applications require different levels of occupancy information because of their spatial and temporal properties [23,24]. Based on previous research, we have suggested an updated categorization of occupancy levels in smart buildings, as shown in Figure 2. This new classification includes the fundamental aspects of presence, counting, location, tracking, activity, and identity. The richness of information in the categorization of occupancy levels in smart buildings typically increases as we move down from presence to identity. However, the relevance and utilization of each level of occupancy data depend significantly on the specific application, with certain levels being more critical than others for achieving desired outcomes.

Regarding the collection of occupancy information, a wide array of sensor types can be employed, including WiFi, Bluetooth Low Energy, radio-frequency identification, ultrasonic sensors, and environmental sensors (such as CO_2_ and temperature detectors). Each type presents its own unique benefits and limitations. For instance, WiFi and Bluetooth Low Energy leverage existing network infrastructures to detect occupancy through signal variations or the presence of devices, but they may raise privacy concerns. Radio-frequency identification excels in precise detection by identifying tagged individuals, yet it necessitates a comprehensive setup [22,25,26,27,28]. Ultrasonic sensors provide a non-intrusive means of detection, but their performance can be influenced by the acoustics of the environment [29]. Meanwhile, environmental sensors such as CO_2_ detectors provide occupancy insights by measuring human emissions, but their delayed response due to CO_2_ accumulation may not accurately indicate immediate occupancy changes [20,30]. Similarly, temperature sensors can monitor environmental conditions but may not directly reflect occupancy without noticeable fluctuations. Despite the advantages offered by these technologies, our study focuses on passive infrared (PIR) sensors due to their straightforwardness, cost-efficiency, and non-invasive nature. This choice emphasizes the balance between detection accuracy and privacy concerns, highlighting PIR sensors as a privacy-preserving option for occupancy information.

**Figure 3:**
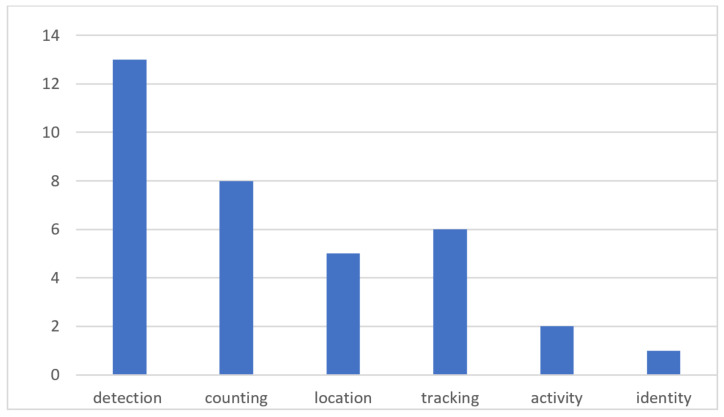
The levels of occupancy information that have been covered in previous literature reviews.

This perspective is particularly relevant in our review of recent literature, which focuses on publications from the past four years and emphasizes the key role of non-invasive sensors in providing various levels of occupancy information. Table 1 displays the reviewed literature reviews on occupancy information. It includes six columns: the first lists the years covered by the reviews; the second describes the main focus of each review; the third details the level of occupancy discussed; the fourth indicates whether machine learning is mentioned; the fifth states whether spatial resolution was considered; and the last column shows that our article is the only literature review article that specifically focuses on PIR sensors for occupancy information. The reviewed literature primarily focuses on sensor functionality, the comparison of sensors, and the applications of occupancy data. Most existing reviews mention machine learning algorithms for non-invasive sensors, but only one article specifically focuses on this aspect [31]. In terms of spatial resolution, all review articles mention it except for literature reviews [26,29,32]. Only two reviews consider all levels of occupancy information. Figure 3 categorizes the review articles based on the levels of occupancy information. The majority of the studies, about 13 literature reviews, look at detection in their work. For counting, location, tracking, and activity, the number of articles is eight, five, six, and two, respectively. Notably, two reviews focus exclusively on counting, with none dedicated solely to the other levels of occupancy information. This indicates that most previous literature reviews concentrate on occupancy detection rather than counting, activity, tracking, location, or identity. Because of that, in our review on PIR sensors, we have decided to look at counting, activity, tracking, and location instead. Furthermore, it becomes clear that for applications such as HVAC control, occupant comfort, health and safety, energy and space utilization, and security, the primary emphasis is on occupancy counting, location, and activity tracking rather than individual identification. This focus is especially relevant considering the privacy issues associated with personal identification. Therefore, our review does not focus on detection and identity. Instead, our attention is directed towards occupancy counting, location tracking, and activity monitoring.

In addition, the previous literature reviews suggest that PIR sensors hold significant potential for capturing occupancy information compared to other sensors. PIR sensors are commonly utilized in settings like homes and offices due to their cost-effectiveness and non-intrusive nature, requiring no pre-existing infrastructure. Although they may have lower accuracy levels on their own, their appeal lies in their ability to improve accuracy when combined with machine learning and other techniques. In contrast to more accurate options like camera-based and wearable sensors, PIR sensors stand out for their affordability, costing typically less than a dollar, and minimal power consumption (around 50 W) [39], making them highly efficient. Moreover, PIR sensors do not capture visual images, reducing privacy concerns associated with intrusive camera-based systems. This combination of affordability, energy efficiency, and privacy protection positions PIR sensors as an excellent choice for straightforward, cost-effective, and non-invasive occupancy detection across various applications.

While some of the previous literature reviews mention PIR sensors, most of them consider these sensors in combination with others, focusing on sensor fusion [22,26]. These reviews typically restrict the use of PIR sensors to mere detection, ignoring their potential to capture other levels of occupancy information, such as activity. Moreover, we observe a notable gap in the existing literature: there is a lack of comprehensive reviews that consider the integration of occupancy information with machine learning techniques in conjunction with PIR sensors. Although these reviews often compare PIR sensors with different sensor types, they do not specifically address the application of machine learning to different levels of occupancy information based on PIR sensors. Additionally, while they do not look at and differentiate signal-based and binary PIR sensors for different levels of occupancy information, key aspects such as the spatial resolution of binary and signal-based PIR sensors and the influence of sensor placement are neglected. Consequently, this literature review is specifically dedicated to investigating the application of PIR sensors in people counting, localization (including tracking and location determination), and activity detection. Importantly, we focus on the impact of machine learning algorithms and various data processing techniques applied to PIR sensors, encompassing both binary and signal-based types. We explore the scope and application of PIR sensors in various settings, such as buildings and individual rooms. Furthermore, we analyze the reliability of PIR sensors for accurately capturing occupancy data. This involves evaluating factors such as sensor placement and determining the appropriate number of both binary and signal-based PIR sensors required for different levels of occupancy information. This comprehensive review aims not only to understand the current state of PIR sensor technology but also to provide insights into optimizing their effectiveness in diverse environments.

## 2. Material and Methods

**Figure 4:**
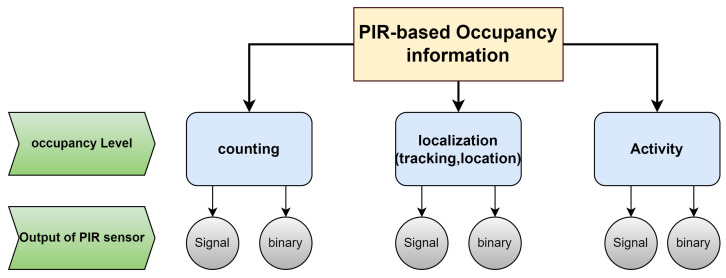
PIR-based occupancy information level.

In our literature review, we have organized the articles into three main categories based on the level of occupancy information: people counting, localization (which encompasses direction, location, and tracking), and activity detection, as illustrated in Figure 4.

We further differentiate these articles based on the type of data output they focus on, whether it be binary or signal-based. This structured approach provides a clear perspective on the varied uses of PIR sensors for capturing occupancy information. In light of our research objectives and the found gaps in previous research, the following four research questions guided our investigation.

### 2.1. Research Questions

- RQ1: Which type of PIR sensor, binary or signal-based, is predominantly used for capturing different levels of occupancy information (counting, localization, and activity)?
- RQ2: What kinds of machine learning algorithms or other methods are mostly used for data processing based on PIR sensors?
- RQ3: What is the suitable quantity and location of PIR sensors within buildings and individual rooms (spatial resolution) to effectively capture different levels of occupancy information, recognizing that this depends on a variety of factors?
- RQ4: What kind of occupant activity can we detect with the binary and signal-based PIR sensors?

By addressing these questions, this review aims to comprehensively explore the applications of PIR sensors at each level of occupancy information sensing, as well as the challenges and advancements in PIR sensor technology for occupancy sensing.

### 2.2. Search Process

To conduct this literature review, we looked at articles from 2015 to 2023 and implemented a structured search strategy. We primarily focused on key scientific databases known for their extensive collections of academic and research papers—namely, Web of Science, Scopus, IEEE Xplore, and Google Scholar. The initial phase of our search process was dedicated to designing and developing a list of keywords and search terms that directly addressed our research questions, as elaborated in Table 2. Each keyword and search term was carefully selected to align with the specific inquiries outlined in our research questions. This design approach ensured that our search specifically uncovered articles relevant to our research objectives. By utilizing the “All fields” filter option within these databases, we were able to thoroughly search through all available metadata—titles, abstracts, keywords, and full texts—to ensure comprehensive coverage of all articles.

**Figure 5:**
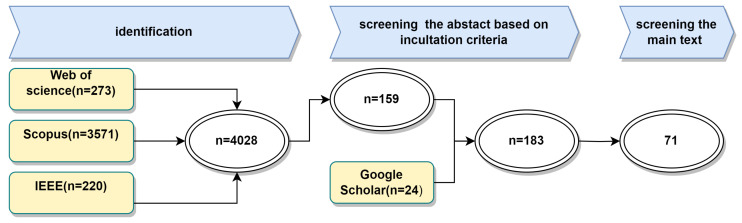
Search methods.

Our extensive search in the mentioned databases led to the discovery of a large number of articles. Specifically, we found 3571 articles in Scopus, 220 in IEEE Xplore, and 273 in Web of Science. As depicted in Figure 5, this summed up to approximately 4028 articles, indicating a rich body of research related to PIR sensors.To refine this large pool of literature, we applied both inclusion and exclusion criteria as outlined in Table 3.

The first step involved removing duplicates, resulting in the exclusion of 83 articles. We then screened the titles and abstracts of the remaining papers based on our inclusion and exclusion criteria, significantly reducing the number of articles to 159. Additionally, we added 24 articles from Google Scholar based on the same criteria, bringing the total to 183 articles relevant to our study. The final step entailed an analysis of the main text of these 183 articles. This in-depth assessment was crucial for evaluating each article’s direct relevance and contribution to our research focus. Following this thorough evaluation, we narrowed our selection down to 71 articles that specially focused on counting, localization, and activity, in line with our inclusion criteria.

## 3. Result

**Figure 6:**
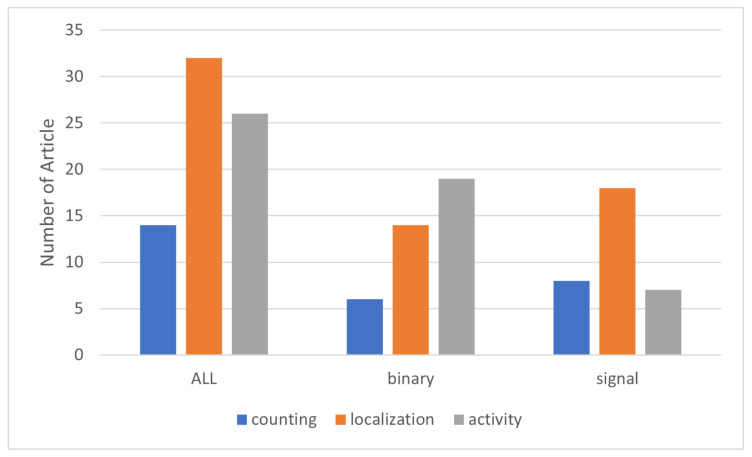
Binary and signal-based PIR for occupancy information.

In this section, we present the results of our literature review. Our analysis indicates that PIR sensors are increasingly utilized for obtaining various levels of occupancy information, with a marked preference for localization and activity detection compared to counting, as shown in Figure 6.

**Figure 7:**
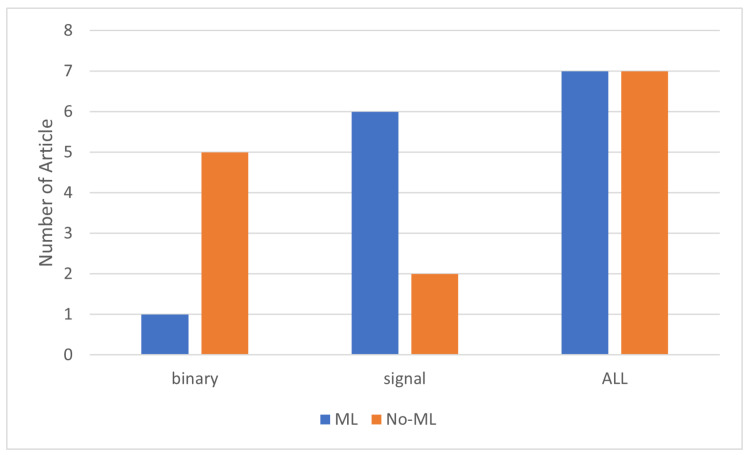
Machine learning with binary and signal-based PIR for people counting.

Based on our findings, the use of PIR sensors for localization and activity detection is more prevalent than their use for multi-person counting. The number of articles using PIR sensors for people counting totaled 14, whereas for localization and activity detection, the numbers were 32 and 26, respectively. Furthermore, the use of both binary and signal-based PIR sensors for people counting and localization is approximately the same. However, for activity detection, binary sensors are twice as popular as signal-based PIR sensors. Additionally, binary sensors are more commonly used for activity detection and localization, whereas signal-based sensors are more favored for localization over counting and activity detection, with about 18 articles focusing on this level. Regarding the use of machine learning with PIR sensors, it is primarily used for occupancy detection. However, the application of machine learning varies at each level of occupancy information detection for both binary and signal-based PIR sensors. Figure 7 shows the use of machine learning algorithms for people counting, highlighting a greater use of these algorithms with signal-based rather than binary sensors, with only one article applying machine learning to binary sensors.

**Figure 8:**
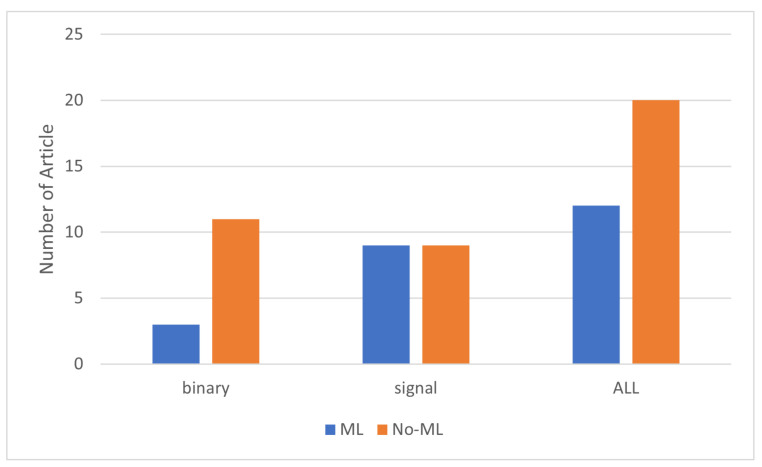
Machine learning with binary and signal-based PIR for localization.

In the context of localization, we found that machine learning is more popular with signal-based PIR sensors, as depicted in Figure 8.

**Figure 9:**
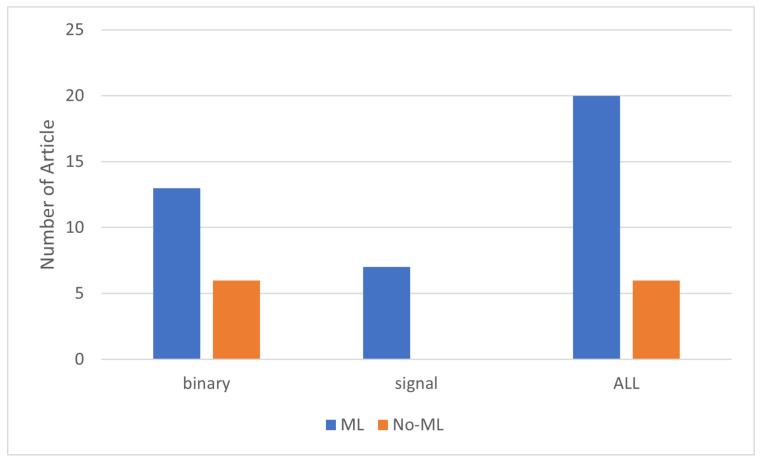
Machine learning with binary and signal-based PIR for activity detection.

Additionally, we found that PIR sensors are widely used in activity detection, noting their significant use in both binary and signal-based sensors. As Figure 9 shows, 20 articles on the use of PIR sensors for activity detection employ machine learning algorithms: all of the articles on signal-based PIR sensors and two-thirds of the ones on binary PIR sensors.

### 3.1. Comparing Binary and Signal-Based Sensors for Occupancy Information

PIR sensors are extensively employed in a variety of applications, including motion detection, security systems [40,41], healthcare [42,43], and energy conservation. These sensors detect infrared light released by objects in their range of vision, which is commonly generated by people and animals owing to their body heat [44]. A PIR sensor is made up of a pyroelectric sensor that detects levels of infrared radiation and a Fresnel lens or mirror that focuses the infrared signals onto the sensor. PIR sensors are divided into two main types: binary and signal-based. Binary-based PIR sensors produce a clear and direct output, indicating ‘1’ when motion is detected and ‘0’ when there is no motion [45,46], and they are often utilized in applications such as turning on/off lights [47] or activating an alarm. On the other hand, signal-based PIR sensors provide a more complicated output, often in the form of an analog signal that fluctuates with the intensity of observed infrared radiation [43]. This signal may give more precise information [48], such as the size or speed of the moving item, making these sensors suited for more advanced applications such as indoor localization or thorough motion analysis. Table 4 compares binary and signal-based PIR sensors in various aspects based on previous research. Notably, the fourth and fifth rows of the table highlight differences in cost and power consumption. Binary sensors tend to be more cost-effective, making them suitable for budget-conscious applications. In contrast, signal-based sensors are more expensive and consume more power due to continuous signal processing, but due to this processing, they are better suited for situations that require precise and detailed motion analysis.

### 3.2. PIR Sensors for People Counting

While PIR sensors are traditionally used to detect whether a space is occupied or not by indicating the presence of at least one person [49,50], this section of our results focuses solely on those studies that examine PIR sensors that detect the presence of more than one person (multi-person counting). We explore advanced methodologies and algorithms that enhance the capabilities of PIR sensors, enabling them to count multiple persons. This exploration offers a deeper analysis of room occupancy detection, illustrating the potential of PIR sensors to provide more comprehensive occupancy data, which is crucial for various applications in smart building management. In Table 5, we present previous studies that utilized signal-based and binary PIR sensors for multi-person counting.

**Binary PIR sensors for counting:** Regarding using a binary PIR sensor for people counting, Hitiyise et al. presented a method for counting using two PIR sensors. The system was tested using Proteus software and real hardware. The method is simple and does not use any machine learning: the count decreases when individuals exit and trigger the inner PIR sensor first, and it increases when they enter and activate the outer sensor first [51]. In this research, detection areas are decreased and just focused on the entrance areas in order to improve the accuracy of the system. In another work, Udrea et al. also developed a people counting system [52] by using two binary PIR sensors. When one sensor detects motion, it checks to see whether the other does as well within a limited period of time (for example, three seconds). If both sensors detect motion within this time period, it is considered an entrance or exit. Following the count, the sensors reset and wait for the next individual. A limitation of this method is its difficulty in differentiating between two individuals entering a location simultaneously. Similarly, in scenarios where individuals congregate at the main entrance upon entering or exiting a building, distinguishing between them becomes challenging. In order to prevent obstructions or false-positive detections, the study by Khan et al. [53] strategically mounted the sensors beneath office desks.

**Table 5:** Previous articles on multi-person counting with PIR sensors.

Ref	Output	Location (Number of Sensors)	Method	Results	People Number	Spatial Resolution
[54]	Binary	Entrance (1), wall (1 in each room)	Own algorithm	Accuracy (86.78%)	6	Building
[51]	Binary	Entrance (1 inside and 1 outside)	Own algorithm	NA	Any	Building
[53]	Binary	Under the desk (1 for each table)	Own algorithm	Accuracy (PIRATES 87.5%)	3	Building
[55]	Binary	Wall, ceiling, door (25-TM004 data set)	Machine learning	Accuracy (Unsupervised learning (sMRT 80%, NN-sg 80%, GNN-sg 83%))	4	Building
[52]	Binary	Entrance (1 inside and 1 outside)	Own methods	No error	Any	Room
[56]	Signal	On bar (3)	Own algorithm	Accuracy (90%)	Any	Specific area
[39]	Signal	Room corner (4)	Machine learning	Accuracy (CNN + BiLSTM 99.5%)	3	Room
[57]	Signal	Entrance (1), wall (1 in each room)	Machine learning	Accuracy (SVM 77.3%)	80	Room
[58]	Signal	Entrance (8 inside and 8 outside)	Machine learning	Accuracy (CNN 92.75%), (DT and RBMLR 60%), (LR 78%), (NB 82%)	any	Room
[59]	Signal	Wall room corner (4)	Own algorithm	Accuracy (80%)	3	Room
[60]	Signal	Wall (1)	Machine learning	Accuracy (hidden Markov models linear regression 99%)	7	Room
[61]	Signal	Robotic actuator (1)	Machine learning	Accuracy (ANN 91%)	3	Room

The primary aim of this research was to assess the effectiveness of using PIR sensors installed beneath the desks of users for gathering extended occupancy information in both open-plan and individual office areas. This technique was implemented in two office buildings over a seven-month period. The system successfully identified the presence and number of occupants with 87.5% accuracy compared to manual counting. Another binary-based people counting method was proposed by Masciadri et al. [54]. It involved a real-world experiment in which an apartment was equipped with eight PIR sensors, one in each room, and a contact sensor on the main door. Each PIR sensor has a 2 s blocking time, requires two movements to activate, and has a 12-s time window for motion detection. The contact sensor, used for monitoring the door and windows, is less complex than the PIR sensors and activates when the main door is opened. This setup provides a comprehensive monitoring system for tracking and estimating the number of occupants in the smart home. It divides sensor data into ‘fragments’ based on movement, such as a person moving from one room to another. The system’s layout is represented as a directed acyclic graph (DAG), which helps in understanding room adjacencies and detecting special events like entries (‘Go in’) and exits (‘Go out’) from the house. An important aspect is the delayed room status representation (DRSRt), where each person in a room is assigned a dynamically changing value based on sensor data, reflecting the likelihood of their presence. The system also employs an inference engine with a confidence score to resolve ambiguities and accurately infer the status of each room. It uses a multi-branch inference approach to handle situations where the number of people cannot be precisely determined, allowing for more accurate occupancy estimations over time. This advanced system is designed to provide a continuously updated and accurate understanding of the occupancy and movement patterns within a smart home environment. The accuracy of this algorithm is 86.78% for a maximum of six people. Giaretta et al. [62] introduced a novel graph-based technique for estimating the minimum number of people in a small building. The method involves mapping the smart home’s sensor network onto an undirected graph, where each sensor is a vertex. The study elaborates on essential concepts such as independent sets, maximal independent sets, and maximum independent sets within this framework. An independent set, where no two adjacent vertices (sensors) are found, signifies a group of sensors that could not have detected the same individual, thereby indicating a minimum count of people based on sensor activation. The maximal independent set expands on this idea, and the maximum independent set denotes the arrangement with the highest number of vertices, providing a theoretical lower bound for the countable number of individuals. This approach enhances sensor deployment optimization and lays a foundation for accurately estimating the minimum number of occupants in a smart home setting.

Regarding using machine learning for binary base sensors for people counting, Wang et al. introduced a novel method for calculation based on a linear Gaussian dynamic model [55]. This approach is notable for its ability to convert raw motion sensor data into structured vectors, capturing both spatial and temporal dynamics inside the home environment. The independence of this technique from ground-truth annotated sensor data or in-depth understanding of sensor layouts is a key benefit. The linear Gaussian dynamic model is used to calculate the probability hypothesis density of the people living there, improving the accuracy of predictions based on sensor data. Notably, the sMRT (multi-resident tracking in smart homes with sensor vectorization) system has been successfully used to monitor residents successfully in an unsupervised way, demonstrating a degree of accuracy equivalent to other systems that need more information. In the particular case of the TM004 dataset, the system obtained an accuracy rate of about 80%.

**Signal-based PIR counting:** Regarding signal-based PIR sensor for people counting, in a study by Liu et al. [59], PIRATES is proposed as a device-free localization system utilizing signals from PIR sensors. The key feature of PIRATES is the extraction of a novel location metric termed “azimuth change”, which relies on the physical properties of PIR sensors. The system achieves 80% accuracy in counting people, and is particularly effective in counting three individuals. Another approach based on signal processing for counting individuals in a specific area is proposed in [56]. Three sensors cover a 3 m wide path. They are mounted 5 m up on a pole and positioned to avoid overlapping fields of view, minimizing double counts. The algorithm developed uses PIR sensors to count pedestrians by measuring the time it takes for a person to move across the sensor’s field of vision (TC) and compares it to the time the sensor signal stays active (TH). If a sensor remains at HIGH for the duration of TH, it implies a person has passed. This count is adjusted to ensure individuals walking closely are not counted as one entity, reducing errors during busy times. The counts from each sensor (PIR1, PIR2, PIR3) are added to get the total number of people over a 15 min period. The accuracy of this method is about 90%.

With regard to using machine learning for people counting, Leech et al. demonstrated the efficacy of a Baysian machine learning algorithm [63]; their methods could predict the number of occupants with a margin of error of ±1 to ±2. Their method, tested in meeting environments with known occupancy, achieved an accuracy of over 80%, efficiently estimating occupancy within a one-person error range. In another work [60], Raykov et al. applied a specific type of machine learning called infinite hidden Markov models (iHMM) that allows effectively using data from a basic PIR sensor to determine the number of people in office. This study also compared three different ways of implementing the iHMM, named beam sampler, Gibbs sampler, and iterative MAP, to see which was best for predicting occupancy from the PIR sensor data. They looked at how these methods performed over various time lengths, from 30 s to 20 min. The results showed that the iterative MAP was not only the most efficient in terms of computation but also required far fewer steps to reach a reliable prediction compared to the other two methods. Another signal-based approach for counting people that uses machine learning is proposed by Tsou et al. [58]. In this work, a system was developed to count individuals, featuring a PIR sensor array that detects people passing by. This system utilizes captured signals to determine the number of people entering or exiting. Eight sensors are installed outside, and another eight are inside the entrance. They record data in roughly 6 s. The PIR array is employed for immediate sensing upon detection of passersby. Various methods, such as convolutional neural networks (CNNs), a combination of restricted Boltzmann machine (RBM) and logistic regression, decision trees, and naïve-Bayes, are used for effective classification with the PIR sensor array. An experimental study tested various classification algorithms on a dataset created using the PIR sensor array to categorize different passing situations. These included CNN, a pipeline of RBM and logistic regression (RBMLR), decision trees (DT), and naïve Bayes (NB). The CNN proved to be the most accurate, achieving up to 92.75% accuracy with all 16 PIR sensors. The accuracy rates for RBMLR and DT were 60%, logistic regression 77%, and naïve Bayes 82%. Another machine learning-based signal approach was introduced in [39]. The approach involves processing signals from multiple PIR sensors through a complex neural network. The input signals are divided into segments, each representing a specific time interval. The network structure is inspired by audio signal separation techniques, utilizing a combination of a 1D CNN layer, two BiLSTM layers with layer normalization, and a fully connected layer. These components effectively separate the signals into individual components, which may represent people or noise sources. A subsequent single-person detection module refines these components to determine if they correspond to individuals. To optimize the model, a permutation-invariant loss function is employed during training. With carefully chosen hyperparameters, this module accurately counts the number of people in a monitored area. In addition, the article demonstrates the effectiveness of preprocessing and data augmentation strategies. The results indicate that various combinations of PP/DA strategies contribute to enhancing PIRNet’s performance, achieving an accuracy of 99.6 for detecting three persons.

A new signal-based method system was developed by Andrews et al. [61]. Central to this innovation is a single Panasonic AMN 24412 PIR sensor, strategically placed in an academic building’s room to optimally scan the area. This sensor is enhanced by a robotic actuator and microcontroller integration for expanded detection and data processing capabilities.The primary role of the MI-PIR system is to reliably determine the number of people in a space, achieving an impressive 91% accuracy. This is accomplished through the employment of an artificial neural network (ANN) which analyzes the sensor’s analog data to accurately assess the occupancy. This feature is particularly valuable given that traditional PIR sensors frequently fail to recognize stationary individuals, causing errors in occupancy counts. As a result, the advanced technique adopted by the MI-PIR system presents a more trustworthy alternative for scenarios like indoor tracking, where precise detection of occupants is essential. The MI-PIR system’s novel approach, therefore, offers a more reliable solution for applications like monitoring indoor environments where accurate people counting is crucial. Regarding determining intervals for people counting instead of counting the exact number of people, in a study by Zhang et al. [57], a single PIR sensor was installed at the entrance of a studio, capable of discerning the direction of both entry and exit. In this research, instead of pinpointing the exact number of people, they used number intervals to classify how many occupants were present. This approach was chosen because, in a large space with many people, a small difference in the exact count of occupants usually does not significantly affect control strategies. However, it is essential to set these number intervals correctly to ensure that deviations stay within acceptable limits. They reviewed many studies on how to count people in different places, noting the types of spaces, their sizes, and how these studies measured results. They used this information to create a system for classifying the number of occupants in their own study, as follows: [0], [1–2], [3–4], [5–6], [7–9], [10–12], [13–15], [16–18], [19–22],…[87–96]. In this research, the midnight reset PIR method has been used. The midnight reset PIR method aims to enhance the accuracy of PIR sensors by addressing error accumulation issues. The strategic reset at midnight ensures a clean slate for the sensors, minimizing inaccuracies and optimizing their performance in detecting human presence during the day. In this research, different machine learning algorithms have been used, and SVM is considered the best algorithm. The combination of PIR + CO_2_ achieves an overall accuracy of around 42.9%, which improves significantly to 85% when recognizing adjacent intervals, and its RMSE value of 1.21 number intervals confirms its reliability. Conversely, the midnight reset PIR method, although simpler, falls short in all three metrics with an accuracy of 36.4% even considering adjacent intervals at 77.3, and an RMSE (root-mean-square error) value of 1.39. In summary, the PIR + CO_2_ method proves to be more effective for accurate occupant counting.

**Figure 10:**
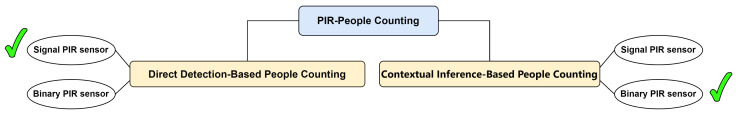
Binary and signal PIR for people counting.

Based on these studies, we have categorized people counting methods, as illustrated in Figure 10.

- **Direct Detection-Based People Counting** In direct detection-based people counting, both signal-based and binary-based methods can be employed to count individuals within a specific area. Advanced signal processing and machine learning are applied to signal-based PIR data for a more accurate count, providing detailed insights with fewer sensors. Conversely, while the binary-based approach can utilize machine learning and other techniques, it generally requires a larger number of sensors to achieve similar accuracy. Overall, the signal-based method is typically preferred for specific areas due to its efficiency and the depth of information it provides, making it a more effective solution for accurately counting people.
- **Contextual Inference-Based People Counting** Contextual inference-based people counting is a method that relies on the context of an environment to deduce the presence and number of people within a specific area. This approach is particularly effective in areas where behaviors and patterns can be predicted and analyzed. It typically includes door monitoring and stationary monitoring, each utilizing both binary and signal-based detection methods.
- **Door Monitoring:** This technique involves placing sensors at doorways to count individuals as they enter or exit. Binary-based detection has been prevalently employed in previous work, especially for rooms, due to its simplicity and effectiveness in scenarios where the presence of more than one person is unlikely at the same time when they pass the door. However, for the main entrances of buildings where multiple people might enter or exit simultaneously, signal-based detection might be a better option. Signal-based methods can provide more detailed information, allowing the system to differentiate between multiple individuals at the same time.
- **Stationary Monitoring:** This method places sensors in areas where people are expected to be stationary, such as under a table or at a desk. Movement detected in these zones is interpreted as an indication of the presence of a person. Both binary and signal-based detection can be used here. Binary-based might suffice in less complex scenarios where any movement is a strong indicator of presence.

### 3.3. PIR Sensors for Localization

In recent years, there has been a tremendous increase in research into human localization with passive infrared (PIR) sensors. PIR sensors are widely used in this field because of their inexpensive cost, low power consumption, and non-intrusive nature. Researchers have largely investigated two major ways to leverage the potential of PIR sensors for localization: binary-based methods and signal-based methods. We categorize previous research based on binary and signal-based methods, as you can see in Table 6.

**Binary PIR for localization:** Some PIR-based localization systems rely on binary data obtained from PIR sensors’ raw output, which essentially informs whether a person is present or not within the sensor’s detection zone. The accuracy of localization using this approach is contingent on finely dividing the detection zones. Put simply, the smaller and more precisely defined these individual zones are, the more precise the localization becomes. However, achieving this high level of precision necessitates deploying a substantial number of PIR sensors and implementing a meticulous deployment strategy. Yang et al. developed a cost-effective and compact system for tracking humans using a network of PIR sensors [64]. When a sensor detects a person, it activates a detection line along the angular bisector of its field of view (FOV). The system is designed for easy calibration, requiring only one sensor node to be calibrated instead of the entire setup, enhancing flexibility with a bearing-crossing location technique. After that, Yang et al. combined this method with region partitioning to pinpoint multiple human locations [65,66], initially using a probabilistic neural network or naive Bayes classifier for rough positioning, followed by the bearing-crossing method for greater accuracy.

**Table 6:** PIR sensor for localization.

Ref	Output	Location	Spatial Resolution	Number of Sensors	Algorithms	Number of People	Machine Learning
[67]	Binary	Floor	Room (specific area)	9 nodes	(Anti-logic) distance error 0.49 m for target 1 and 0.50 m for target 2.	2	No
[68]	Binary	Floor	Room (specific area)	9 nodes	(Own methods), average distance errors target 1 (0.62 m) and target 2 (0.53 m)	2	No
[69]	Binary	Wall	Room	16	(Own methods (graph based)), average tracking error is 0.4706 m	2	No
[70]	Binary	Corner	Room	4	(Own methods (ULT)), RMSE values of 0.33 m for ULT and 1.09 m for RDLT	1	No
[71]	Binary	Ceiling	Buildings	10	(Own methods–Maps and A-Star Algorithm), average error (m) 0.21	1	No
[72]	Binary	Ceiling	Building	9	(Own methods–map based), position error 0.6 m	1	No
[66]	Binary	Floor	Room (specific area)	9	(NBCL-Naive Bayes classifier), distance error (m) target 1 (0.67) target 2 0.56	2	Yes
[73]	Binary	Ceiling, Wall	Buildings	7	(Own methods–Transferable Belief Model), scenario01 Error rate 3.3%	1	No
[74]	Binary	Robot	Room	4	(Decision Tree classifier), accuracy 83.3%.	1	Yes
[65]	Binary	Floor	Room (specific area)	9 nodes	(Probabilistic neural network CLBNNRP) RMSE (m) X (33.5) Y (0.33) Distance (0.33)	2	Yes
[75]	Binary	Floor	Room (specific area)	9 nodes	(Own methods), Credit-Based Method average distance errors (0.47 m) target 1 target 2 (0.42 m)	2	No
[76]	Signal	Ceiling	Room	4	(ANN + LSTM) Mean Error (m) 0.68	2	Yes
[77]	Signal	Corner	Room	4	(CNN, BiLSTM) accuracy 96.1%, average localization errors 0.82 m	3	Yes
[78]	Signal	Wall	Room	4	(CNN, BiLSTM) average localization errors 0.88 m	3	Yes
[59]	Signal	Wall	Room	4	(PIRATES) average localization error 0.87 m	2	No
[39]	Signal	Wall	Room	8	(CNN, BiLSTM), f1 Score 0.94, average localization errors 1.34 m	5	Yes
[79]	Signal	Ceiling	Room	5	Mean distance error (CNN-LSTM 0.2359), (CNN-BiLSTM 0.3131), (CNN-GRU 0.5198) Particle filter (0.5482 m)	1	Yes
[80]	Signal	Ceiling	Room	5	Mean distance error CNN-LSTM (0.2379) CNN-BiLSTM (0.3222) CNN-GRN (0.5172) SVR (0.69)	1	Yes
[81]	Signal	Ceiling	Room	4	(Own methods (signal-based)), Mean error (m) 0.7, accuracy 80% or higher in all scenarios	1	No
[82]	Signal	Ceiling	Room	2 module (4 PIR in module)	(Own methods), RMSE (meter) (Localization Algorithm) 0.3118 (Kalman Filter) 0.285	1	No
[83]	Signal	Ceiling	Room	5 module (9 PIR in module)	(own methods–Kalman Filter), RMSE 0.68	1	No
[84]	Signal	Ceiling	Room	4	(own methods–Kalman Filter), 0.254, TBM-based Hybrid Method 0.219	1	No
[85]	Signal	Wall	Room	2 Sensing Tower 16 PIR for each	accuracy of 0.113 m	1	No

**Figure 11:**
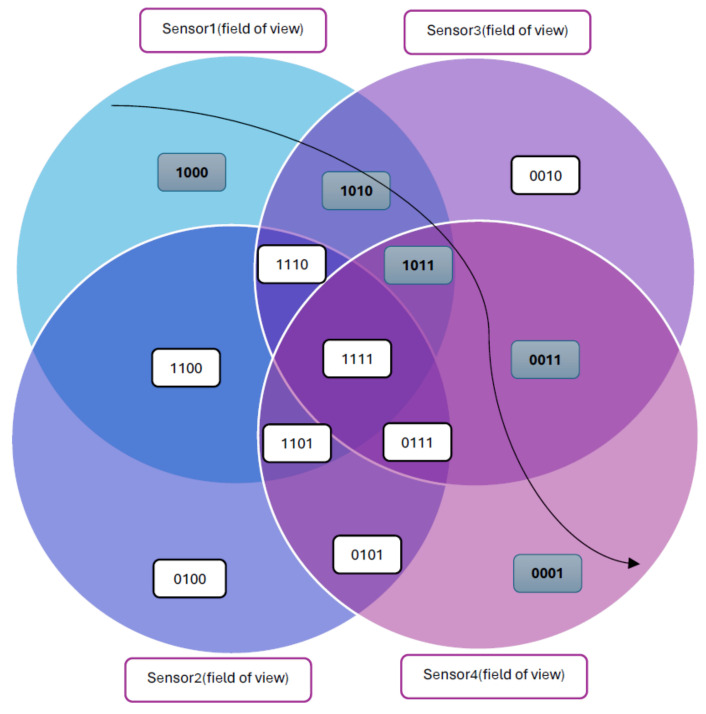
Space encoding in [69].

Then, Yang et al. introduced a credit-based clustering and location (CBCL) method for locating multiple humans [75]. This method innovatively assigns credits to measurement points based on their probability of being within the sensor’s field of view, prioritizing the most likely human locations. The credit-based system improves location tracking accuracy by 40%, reducing errors to 0.24 m compared to the previous 0.40 m. Furthermore, in multiple target simulations, the CBCL method not only reduced execution time by 21.4% but also increased location accuracy by 29.9% and 14% for two separate targets, respectively. These results demonstrate the CBCL method’s superiority in both accuracy and efficiency over previous methods, representing a significant advancement in PIR sensor-based human tracking technology. This method assigns credits based on the frequency of a crossing point appearing within the sensors’ activated regions, selecting only those with the highest credits for positioning. This approach speeds up the process and allows more flexible node placement, but it does not account for targets outside a sensor’s range. Additional logic was needed to retain some lower-credit measurement points near a target. To address this, Yang et al. proposed an anti-logic bearing-crossing algorithm [67] that first identifies points falling outside active regions, then inverts this to maintain all high-credit points, enhancing effectiveness for various targets and sensor placements. They introduced a refined credit-based algorithm for accurately determining effective measurement points for indoor multi-person tracking, alongside a dynamic pruning algorithm to allocate these points to respective targets. This innovative system, validated through simulations and experiments, offers a significant advancement in accurately tracking and locating multiple individuals, making it particularly valuable in surveillance and security applications. Mean distance error for this method is about 0.45 m. To enhance the credit location algorithm for accurately obtaining measurement points essential for tracking multiple humans indoors, Yang et al. devised a dynamic pruning algorithm that allocates all effective measurement points across various targets [68]. This novel approach simplifies and enhances the accuracy of tracking multiple human targets. The improved credit method efficiently retains all effective measurement points without needing extra logical judgment, making it more intuitive and less complex than previous methods. The dynamic pruning algorithm further refines this process by reducing the number of measurement points and accurately assigning them to targets based on predicted positions, effectively transforming the challenge of multiple human location tracking into a simpler task of single human tracking. This method incorporates advanced algorithms such as the Kalman filter and IMM tracking, representing a significant leap in the precision and reliability of human indoor location systems. In another binary-based method for tracking multiple people, Lu et al. conducted research for tracking several humans by using distributed (PIR) sensor networks [69]. Their main emphasis was on enhancing sensor selection and calibration. The study presents a sensor selection method based on information gain that aims to pick sensors that optimize the mutual information between sensors and targets. This technique efficiently balances the accuracy of tracking and the efficient use of resources. Furthermore, a sensing probability model is used to calibrate the sensors, which is essential for precise monitoring of many targets. This approach performs sensor calibration by analyzing the probability of detecting a target in segmented spatial grids, then adjusting the placements and orientations of the sensors accordingly. In addition, the research employs a factor graph-based message-forwarding mechanism to improve the accuracy and efficiency of the tracking process. This technique, which has been validated by simulations and tests, represents a notable progression in the area of distributed sensor networks for human monitoring. The main idea of this research is based on space encoding for three binary PIR sensors as shown in Figure 11.

Lu et al. proposed another method based on encoding, which introduces a framework for tracking multiple targets using distributed binary sensors [86]. The aim is to minimize data throughput while maintaining accurate tracking. The framework involves space encoding using a low-density parity-check matrix and measurement decoding through linear programming and Bayesian estimation. Key challenges addressed include efficient measurement representation, reducing localization errors, and optimizing binary sampling geometries. The paper validates its approach through simulations and experiments, demonstrating effective multitarget tracking with minimal data requirements. Zade et al. suggest another binary-based localization method that compares a unique methodology for target tracking in PIR sensor networks with the traditional particle filters (PF) technique [70]. This new method is applied across several motion models, including uniform linear trajectory (ULT), random direction linear trajectory (RDLT), random direction backward turn linear trajectory (RDBLT), and horizontal motion linear trajectory (HMLT), each representing different target movement patterns. The study demonstrates that the new method yields better tracking accuracy and convergence, especially in dynamic scenarios, as evidenced by lower RMSE values such as 0.33 m for ULT and 1.09 m for RDLT. This improved performance, particularly in complex motion models like RDBLT and HMLT, highlights the method’s efficiency and reduced computational complexity compared to PF. In recent research, which concentrates on binary output and machine learning applications, Ciuffreda et al. have integrated a robot to improve localization techniques [74]. This research focuses on the indoor localization of elderly individuals using a mobile social robot equipped with multiple PIR sensors. Despite the challenges posed by overlapping detection fields in the multi-sensor setup, the system is designed to accurately detect human presence. A pivotal element of their methodology is the application of a decision tree classifier algorithm trained to differentiate between various scenarios and improve localization accuracy. The outcomes of their study indicate high accuracy levels, reaching 96% in controlled environments with constrained movements. Nonetheless, the accuracy slightly diminishes to 83.3% in less controlled conditions. This demonstrates the system’s effectiveness across a spectrum of indoor scenarios, suggesting a promising avenue for elderly care via non-invasive and cost-effective technology.

Regarding location tracking based on binary PIR sensors in entire buildings, Fanti et al. present a framework for the strategic placement of binary sensors in indoor environments to improve the tracking of inhabitants, particularly in the context of ambient assisted living (AAL) [87]. This framework aims to optimize sensor placement for effective monitoring while considering the physical layout of the environment and obstacles like furniture and walls. It utilizes an integer linear programming model to balance coverage precision and environmental constraints. In another study on building scale, Yang et al. presented an advanced building-based method for indoor human localization combining PIR sensors with an accessibility map, which was proposed and tested through simulations [72]. This hybrid approach involves tracking an individual’s routine movements within a home, such as arriving from work, visiting different rooms, and engaging in various activities. The accuracy of this method was assessed using the cumulative distribution of absolute position errors. The results indicated a significant improvement over the PIR-only method; the hybrid method achieved a 95 percent accuracy probability of position errors within 0.6 m, compared to 0.8 m for the PIR-only approach. Furthermore, the minimum localization error was reduced to 0.1 m with the hybrid method from 0.2 m with the PIR-only method. This demonstrates the effectiveness of integrating PIR sensors with an accessibility map in enhancing localization accuracy within indoor environments. Yang et al. integrate their methods with a grid-based accessibility map and the A-star algorithm [71]. The grid-based accessibility map represents the environment as a grid, where each cell indicates the probability of a person’s presence, considering furniture locations and human visiting habits. The A-star algorithm, a best-first search method, uses this map along with PIR sensor data to optimize tracking. It calculates the best path using a cost function based on distance and heuristic estimates. The results from their experiments, which involved predefined trajectories in a mock apartment, demonstrated the effectiveness of this method. The maximum distance error recorded was 0.747 m, and the minimum was 0.021 m, with an average mean distance error of 0.227 m for one route. Another route showed a mean distance error of 0.188 m. These results were compared with recent human tracking projects, indicating competitive average error rates and overall accuracy. This study illustrates the potential of combining a grid-based accessibility map with the A-star algorithm for efficient and accurate indoor human tracking using PIR sensors. Henni et al. successfully enhanced their previous multiplex binary localization approach in the scope of building [73]. This enhancement was aimed at resolving the ambiguity in detecting transitions between zones, achieved through the implementation of a filtering technique based on the transferable belief model (TBM) instead of the traditional Bayesian framework. A significant aspect of this work was the development of a new method for choosing an appropriate discounting factor. This factor is crucial for weighting information sources relative to each other, based on the Dempster–Shafer normalization function. The study efficiently covered the desired zones of interest with a reduced number of sensors, utilizing an overlapping multiplex structure. This novel method demonstrated robustness against fleeting sensor faults, thanks to its filtering capabilities, and effectively managed reasonable uncertainties in the sensors’ field of view, which are critical during transitions between zones. The methodology’s efficacy was validated through experiments using commercial single PIR sensors with modified fields of view, showcasing the advantages of the TBM method compared to the previous Bayesian approach.

**Signal-based PIR sensors for localization:** Signal-based methods are more popular for localization because they can receive more information from the signal. In the study by Narayana et al. [88], the researchers explore the previously overlooked potential by examining the analog signals from an array of PIR sensors. This novel method enables the enhanced functions of PIR sensors, traditionally limited to binary detection tasks, to be fully utilized. By retaining and analyzing the richness of the analog signals, the research achieves accurate object classification and localization, demonstrating significant precision up to 5 m. Complementing this advancement, another study proposes a novel analog-based system for room localization [85]. This system employs rotating and modified PIR sensors with analog outputs. Such an advancement significantly improves the accuracy of distance estimation between the device and human targets. The implemented prototypes demonstrate that the system can achieve an accuracy of 0.113 m in a 12 m × 6 m area, highlighting its potential for accurate and real-time tracking in various applications. Luo et al. propose another signal-based localization system [83]. The system consists of five sensor nodes, creating a wireless network, each equipped with a unique reference structure for field of view (FOV) modulation. This configuration facilitates effective spatial segmentation by decoding spatial data through a coding scheme that segments the monitored area into various sampling cells. The localization algorithm employed incorporates a Kalman filter and smoother to refine the accuracy of human target location estimates by processing data streams from different sensor nodes. Furthermore, the system emphasizes the extraction of signal features, with a particular focus on the short-time energy metric, to capture the variation in energy of the PIR sensor signals. When tested in a real office environment, the system achieved an impressive average root-mean-square error (RMSE) of approximately 0.6 m while tracking a single human target moving at various speeds. This innovative approach represents a significant advancement in the field of human indoor localization, especially in attaining high spatial resolution and tracking accuracy with PIR sensors. Lai et al. proposed another method based on the Kalman filter, developing a novel indoor localization system using two passive infrared (PIR) sensor modules [82]. This approach involved the analysis of analog signals produced by human movement within a coverage area segmented into nine discrete cells. The system utilized a Kalman filter for localization, a technique celebrated for its proficiency in accurately estimating dynamic systems from incomplete and noisy data. The effectiveness of this system was measured using the (RMSE) metric. The RMSE for the localization algorithm was found to range from 0.3118 to 0.846 m, and the Kalman filter achieved an RMSE between 0.285 and 0.6804 m. These results demonstrate that the system not only simplifies the design of indoor localization systems but also enhances accuracy. To enhance the localization system based on the Kalman filter, a new method was introduced by Wu et al. [84]. This method refines the system’s performance by integrating the Kalman filter, a model-based approach, with a transferable belief model (TBM), a belief-driven strategy, into a novel hybrid approach. This innovative strategy enhances tracking accuracy and stability by leveraging TBM outputs in the Kalman filter’s estimation processes. The researchers conducted a series of three experiments: validating the parameters, conducting a qualitative analysis of TBM tracking, and performing a quantitative study involving both TBM and Kalman filter tracking. The results indicated significant improvements in system stability and positioning accuracy, especially with the hybrid approach. This research significantly contributes to the field of indoor localization by providing a framework that utilizes PIR sensors in a networked setup for efficient and privacy-respectful human localization and tracking. In the most recent research on Kalman filter-based localization presented by Yunus et al. [89], the study introduces an innovative method for tracking humans across various indoor environments. By employing PIR motion sensors coupled with Kalman filter-based estimation, the approach notably enhances accuracy. The findings indicate a decrease in the maximum error for tracking trajectory from 0.28 m to 0.19 m and a reduction in the average error from 0.10 m to 0.07 m.

Another signal-based localization system, which focuses on PIR sensors, introduces a new technique for real-time tracking utilizing these sensors [81]. The primary aim of this approach is to detect changes in azimuth by mainly utilizing raw data from PIR sensors to derive position information based on these alterations. The configuration consisted of four PIR sensors arranged within a 7 m × 7 m area. The collected data was wirelessly transmitted to a computer for analysis, employing a particle filter technique. Through experiments in six different movement scenarios, the system demonstrated an average localization error of around 0.63 m, underscoring its capability to accurately track objects in real-time across a range of situations. Following this research, Liu et al. employ a combination of signal processing techniques (PIRATES), including the use of differential heat flux (DHF) and inverse filtering, to enhance accuracy and robustness against environmental noise [59]. It also incorporates a particle filter algorithm for real-time localization, adaptable to both single-person and multi-person scenarios. This novel approach reduces the need for extensive sensor deployment, making PIRATES more efficient and versatile in various environmental conditions. In a study examining the effect of PIR sensor deployment on localization accuracy, it was found that both the number and placement of sensors significantly impact the system’s precision. The experiment involved various configurations, including single and multiple sensors placed in different geometric layouts. Results showed a clear trend: an increased number of PIR sensors led to improved localization accuracy. Additionally, uniformity in sensor distribution played a crucial role. Configurations with evenly spaced sensors, especially those covering all area angles (as in a scenario with four sensors at each corner), resulted in lower localization errors compared to non-uniform arrangements, such as sensors aligned in a single line. This data is critical for optimizing sensor deployment in practical applications like security systems or smart environments, where precise localization is paramount.

In the context of using machine learning for localization based on signal-based PIR sensors, Yang et al. introduce device-free localization (DFL) utilizing PIR sensors as a cost-effective, low-power, and privacy-preserving method to locate people using a deep learning approach called PIRNet [77]. The newly developed neural network effectively manages multi-person scenarios using two modules: one for counting people and another for locating them. Through these methods, the deployment density of traditional PIR-based approaches has substantially decreased by about 76% while maintaining high localization accuracy. However, there is still considerable potential for improvement in PIRNet, especially regarding the cost of training. PIRNet is based on the assumption that the deployment of PIR sensors in the test environment replicates that of the training environment. If this is not the case, PIRNet requires retraining with additional data gathered from the environments of its new deployments. A new deep-learning-based approach called DeepPIRATES is introduced by Yang et al. [78]. Unlike PIRNet, DeepPIRATES needs only a single training session and then effectively operates in any deployment strategy environment. Additionally, DeepPIRATES retains the benefits of PIRNet, such as low deployment density and high localization accuracy. This method uses a two-step process: deep learning to estimate relative locations from a single sensor, and a particle filter for inferring absolute locations. This method achieves low localization errors (0.55 m, 0.73 m, 0.88 m for one, two, and three-person scenarios) with a low sensor density. Future improvements may include enhancing the particle filter with neural network-based motion models to further increase localization accuracy. This deep learning method, which was also used in [39], shows a PIR sensor-based multi-person localization module that focuses on combining deep learning techniques with domain knowledge. Using a network with layers like 1D convolution and BiLSTM, it processes sensor data to predict positions. The accuracy is measured using a loss function based on the Euclidean distance between the predicted and actual positions. Tests showed that with four sensors, the F1 scores in scenarios with up to five people exceeded 85%. Increasing the number of sensors to six and eight improved F1 scores to over 90% and 93%, respectively, demonstrating the method’s potential for practical multi-person localization applications. Chen et al. present a novel approach known as PIRILS (pyroelectric infrared indoor localization system) [76]. It utilizes a cutting-edge algorithmic structure, primarily based on an artificial neural network (ANN), for indoor multi-target localization. This system integrates a long short-term memory (LSTM) model, enhancing the ANN’s ability to process sequential data from PIR sensors. Additionally, a permutation-invariant strategy is employed to maintain consistency in localization outcomes despite variations in sensor signal order. To further refine its accuracy, the system adopts a data augmentation strategy, enriching the training dataset to better handle diverse motion patterns. This comprehensive algorithmic approach, combining ANN, LSTM, permutation invariance, and data augmentation, enables PIRILS to accurately track and localize multiple targets in indoor environments, showcasing a significant advancement in the application of PIR sensors augmented by deep learning techniques. Ngamakeur et al. introduced a different deep learning-based method for people localization in their study [80], employing CNN and LSTM networks. The process begins with preprocessing the PIR sensor data. The CNN extracts spatial features from this data, identifying patterns and characteristics in the signals. The LSTM analyzes temporal patterns, crucial for understanding time-dependent features in the sensor data. By integrating CNN for spatial feature extraction and LSTM for temporal analysis, the model learns to accurately correlate sensor signals with indoor locations. Their findings demonstrate that the proposed method can adeptly navigate complex situations, achieving an average distance error of 0.23 m, with 80% of the distance errors falling under 0.4 m. Ngamakeur et al. also looked at different deep learning models, like CNN, RNN, and their hybrids, to classify locations and figure out their 2D coordinates [79]. The key architectures evaluated were 1D-CNN, TCNs, LSTM, Bi-LSTM, GRU, and particle filtering. These were assessed using metrics like accuracy, recall, precision, F1 score, and Kappa score for classification, along with mean distance error for coordinate estimation. The study achieved an average accuracy of 77%, with Bi-LSTM-based models demonstrating superior performance. In 2D coordinate estimation, the CNN-LSTM combination emerged as the top performer, with a mean distance error of 0.2359 m. Even though there were some problems, like signal ambiguity and strange signal patterns, the study showed that deep learning could be used for indoor PIR-based localization and tracking. It was very accurate, and it set the stage for future improvements in system scalability and dataset diversity.

Regarding the detection of the direction of people, A novel solution using regularized K-SVD dictionary learning for PIR sensor-based ambient assisted living systems, focusing on detecting human movement, is introduced by De et al. [90]. The proposed modified algorithms, MRK-SVD and MRAK-SVD, show significant improvements in performance compared to existing methods. Another paper by De et al. introduces an innovative approach for detecting human movement direction. They employ a label consistency-based modified sequential dictionary learning method integrated with PIR sensor technology [91]. Their research enhances detection algorithms to handle larger databases more effectively. Their LC-MCAS-DL method is shown to be better than previous MMCP- and MRAK-SVD-based methods, especially in difficult everyday situations like finding intruders. This comprehensive study, supported by detailed experimental data from an advanced PIR sensor system, establishes a new benchmark in the field of human movement direction detection. In a study by Yun et al. [92], classical machine learning and a simple deep learning model for human movement detection using analog PIR are evaluated. Classical machine learning excels in real-time detection, while deep learning achieves approximately 90% accuracy in direction detection with minimal data and scaling benefits. Another method using a PIR sensor is presented by Yun et al. [93]. In this work, a system for counting and direction detection of moving people is employed, employing convolutional neural networks (CNNs) and generative adversarial networks (GANs). Data collected from scenarios involving one to four subjects is utilized. A unique time sequence sensor data augmentation algorithm, the auxiliary-classifier conditional GAN, is developed to improve performance in multi-person movements. It enhances the model’s ability to handle complex scenarios of multiple individuals moving simultaneously. The results indicate significant improvements in counting accuracy, with increases of 7.9%, 9.7%, 26%, and 37.5% for groups of one to four subjects, respectively, compared to the original model without augmentation. In the most recent work by Umutoni et al. [94], a new approach is used to find moving objects with limited real-time resources. This approach uses analog PIR signals as data input and the growing TinyML technology. It achieves a performance accuracy of 80.8%, with the potential for enhancement over time through the application of reinforcement learning.

**Figure 12:**
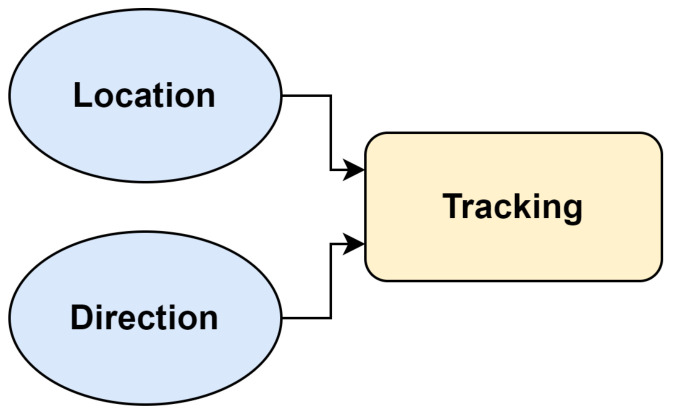
Localization system.

Based on these studies on localization, both signal-based and binary-based systems play crucial roles in determining the direction, location, and tracking of objects or individuals. Signal-based PIR sensors provide a continuous range of values, offering detailed information for localization. This data is instrumental in accurately determining the direction of movement and precise localization by analyzing the intensity and patterns of the signals over time. On the other hand, binary-based PIR sensors, which only indicate the presence or absence of motion, are simpler but can be effectively used for tracking when multiple sensors are networked together, separating the space based on the field of view. By strategically placing these sensors and interpreting the sequential triggering, one can deduce the direction of movement and approximate location. For tracking people using both binary and signal-based sensors, understanding location and direction is essential. This means that effective tracking requires precise knowledge of both where a person is (location) and where they are heading (direction). As depicted in Figure 12, these two elements are fundamental in accurately monitoring movements, allowing the system to provide real-time updates and predict future positions. This synergy between location and direction forms the core of a robust tracking system, whether it is utilizing the detailed data from signal-based sensors or the simpler presence/absence detection of binary sensors.

**Figure 13:**
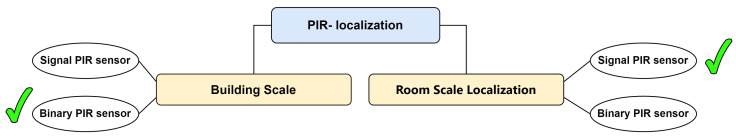
Localization based on PIR sensor.

Additionally, based on previous research, we can categorize PIR-based localization systems into two main types: building-scale localization and room-scale localization, as illustrated in Figure 13.

- **Building Scale Localization:** This level focuses on identifying the specific room a person occupies within a building. Binary-based PIR sensors are the preferred choice for this task due to their efficiency in detecting whether individuals are present or absent as they move between rooms. Positioned at critical points like doorways, these sensors give a broad yet effective overview of where people are located throughout the building.
- **Room Scale Localization:** For pinpointing an individual’s exact position within a particular room or area, signal-based PIR sensors are primarily employed based on extensive research. These sensors provide a continuous stream of data vital for accurately identifying a person’s precise location in a confined space. They analyze the intensity and fluctuations of signals over time, allowing the system to detect subtle movements and specific positions within the room. This detailed approach offers a greater depth of understanding about an individual’s location and movement on a smaller scale compared to more general building-scale localization.

### 3.4. PIR Sensors for Activity Detection

PIR sensors are critical components in the field of activity detection, particularly in smart homes. Binary and signal-based techniques are both used in this area of research, showing the variety of methods that are used. Researchers have developed sophisticated systems utilizing innovative methods to accurately interpret human activities. Machine learning algorithms, deep neural networks, and collaborative reasoning have all been added to activity recognition systems to make them much more useful. These advancements not only enable discreet monitoring but also meet the growing demand for intelligent and context-aware home automation. We summarize binary PIR sensor approaches for activity detection in Table 7.

**Binary-based PIR sensor activity detection:** Based on studies, the utilization of binary PIR sensors for activity recognition has become prominent in smart home applications. Kashimoto et al. [95] present a low-cost, device-free activity recognition system using energy-harvesting PIR and door sensors for smart homes. These sensors, powered by solar panels, eliminate the need for battery replacements. The system recognizes various home activities, such as eating, cooking, and sleeping, by analyzing sensor data. It employs random forest machine learning algorithms for accurate activity classification. The system’s effectiveness was validated in a smart home setting with an average F-measure accuracy of 62.8%. This approach offers a practical solution for monitoring daily activities in homes without intruding on privacy or incurring high costs. Lameski et al. navigated challenges in designing a non-invasive ambient assisted living (AAL) system with PIR for activity recognition in nursing homes [96]. They addressed issues like limited data collection from PIR sensors, the need for easy deployment, and the importance of user-friendly AAL systems for vulnerable populations. In another study, Zhang et al. utilized PIR sensors and machine learning for residential occupant monitoring [104]. They implemented a PIR sensor array to collect data over 71 days, classifying occupant activities into heavy, medium, light, and resting categories, plus differentiating between human and pet movements. Utilizing an optimized support vector machine model, they achieved high accuracy rates (99.7% for training, 90.9% for testing) in identifying activity intensities and locations. The study underscores the potential of PIR sensors in smart homes for precise, privacy-conscious occupant behavior monitoring.

Utsumi et al. present a revolutionary method for early pre-frailty detection, which uses binary PIR sensors to assess walking speed [112]. The system measures the time of passage between two PIR sensor points, enabling continuous monitoring of walking speed in daily living. This real-time assessment allows for the quantitative calculation of the subject’s physical activity. The system’s capability to detect changes in walking speed provides a means to estimate pre-frailty at an early stage. Furthermore, the order and frequency of PIR sensor detections contribute to generating movement routes, offering insights into subject behavior patterns and functioning as a monitoring system.

Most research utilizing binary-based PIR sensors employs the Aruba dataset, which is associated with activity recognition and smart homes. This dataset consists of sensor readings collected from a smart home environment where a variety of sensors, including motion sensors, temperature sensors, and contact sensors, are installed throughout a residential space. These sensors gather data over time, documenting the daily activities and movements of the inhabitants. In this literature review article, our attention is specifically on those studies that exclusively use motion sensors, disregarding any other type of sensor. Fahad et al. developed an activity Recognition by clustering-based classification (AR-CbC) model using PCA for feature selection and Lloyd’s algorithm for clustering activity instances in smart homes [113]. The model applies ET-KNN for classification within clusters, enhancing accuracy, especially for overlapping activities. Tested on Aruba and Kasteren datasets, AR-CbC showed superior performance in precision, recall, and F1 score compared to other classifiers. In Aruba, it achieved 79.65% precision, 76.46% recall, and 91.40% accuracy. In Kasteren, precision and recall were as high as 96.26% and 95.07%, demonstrating the model’s effective activity recognition. Gochoo et al. developed a novel deep convolutional neural network (DCNN) based on the Aruba dataset for four activities [97]. The study converted the annotated binary sensor data into binary activity images corresponding to the activities. Subsequently, these activity images were utilized for training and testing the DCNN classifier. Finally, the classifiers were evaluated using a 10-fold cross-validation method. Experimental results showcased that the best DCNN classifier achieved an impressive accuracy of 99.36%, demonstrating its proficiency in identifying basic activities such as bed to toilet, eating, meal preparation, and relaxing. In another work, Gochoo et al. [98] focused on using DCNN to detect a wider range of activities. The study demonstrates the effectiveness of the DCNN model, achieving high F1 scores of 0.79 for ten activities and 0.951 for eight. The model exhibits exceptional capability in recognizing and differentiating between various activities. This research is pivotal as it offers a non-invasive, privacy-respecting tool for monitoring elderly individuals and sets a path for future enhancements, such as integrating LSTM with DCNN for more comprehensive real-life applications. In their exploration of elderly travel patterns, Gochoo et al. employed diverse machine learning algorithms [98]. The study aimed to discern various movement patterns—direct, pacing, lapping, or random—as indicative of the resident’s cognitive state and potential early signs of dementia. The results, expressed in accuracy percentages for different algorithms, are as follows: naïve Bayes (82.51%), one vs. rest (90.46%), KNN (93.25%), decision tree (93.58%), SVC (93.81%), gradient boost (94.06%), random forest (94.48%), and DCNN (97.84%). The DCNN model demonstrated high accuracy, significantly outperforming classical machine-learning classifiers in accurately detecting and interpreting elderly travel patterns. In a study by Rajesh et al. [102], DCNN algorithms were also employed to detect a broader range of activities, specifically 12 activities. The system underwent evaluation using the Aruba dataset, and the results for detecting these 12 activities showcased a high F1 score of 0.82. The proposed DCNN achieved an impressive accuracy of 98.68%. The study by Xu et al. proposes a novel two-layer multi-granularity activity recognition model [103]. This framework includes a coarse-grained subsystem for recognizing easily-confused activities and a fine-grained subsystem employing machine learning or deep learning classifiers for detailed activity identification. The model, validated using the Aruba dataset, demonstrates superior performance with the two-layer framework and marker-based stigmergy. The accuracy for AdaBoost is 98%, and for DCNN, it is 95%. Despite limitations in DCNN performance due to sample size, the combination of DCNN, the two-layer framework, and stigmergy effectively characterizes spatio-temporal properties. Additionally, the study explores the impact of standard deviation parameters on classification performance, revealing minimal influence from changes in the adjustment coefficient of diffusivity. It notes that accuracy initially increases and then decreases with rising adjustment coefficients of volatility for both machine learning and deep learning classifiers. Continuing this line of work, Xu et al. introduced an innovative technique for event-driven daily activity recognition (DAR) in elderly health monitoring in their study [110]. Their approach utilizes marker-based stigmergy and a directed-weighted network (DwN) to construct a stigmergic activity pheromone trail (APT). DwN outperforms APM, showcasing its superiority in recognizing activities of daily living (ADLs) and reducing activity confusion. Evaluation with the Aruba dataset highlights DwN’s effectiveness, further enhanced by incorporating location information for improved DAR performance, especially in scenarios with clear directionality. The system, featuring AdaBoost or DCNN classifiers, demonstrates robust performance validated through ablation experiments. While diffusion parameters minimally impact DAR, careful selection of volatilization parameters is crucial. Overall, this approach outperforms state-of-the-art methods, emphasizing its efficacy in real-world scenarios. Tan et al. created another deep learning-based technique that makes use of recurrent neural networks (RNNs) [101]. The study employs bidirectional long short-term memory (Bi-LSTM) neural networks and fully connected neural networks (FCNNs) for feature extraction and activity classification. By incorporating external features such as previous activity and begin time-stamp, the model enhances accuracy in recognizing daily activities, demonstrating an F1 score of 0.917, which is a notable improvement over state-of-the-art models. In study by Hwang et al. [105], a new model using deep learning was developed, emphasizing causality feature extraction and fuzzy temporal windows (FTWs) for better precision. The model, tested on Aruba, Cairo, and Milan datasets, effectively distinguishes between easily-confused activities and manages unlabeled data challenges. It employs deep long short-term memory (LSTM), 2D convolutional neural networks (CNNs), and hybrid models for learning spatiotemporal dependencies. The study reported significant improvements in macro-F1 scores, demonstrating the model’s effectiveness in recognizing complex human activities.

Regarding the use of another technique, the study by Jarraya et al. presents a novel approach named distributed collaborative reasoning (DCR) [107]. This approach leverages a multi-agent system where agents with different classifiers observe sensor data, make local predictions, and collaborate for activity identification. This paper introduces an enhanced version, DCR-OL, incorporating online learning where agents learn from their interactions to improve performance. Tests on the Aruba dataset show that both DCR and DCR-OL are more accurate, have better F measures, and have better G means than existing centralized and distributed methods. In [99] by Yatbaz et al., an innovative method for monitoring elderly individuals living alone is proposed through scanpath trend analysis (STA), adapted from eye movement trend analysis. This approach, utilizing binary sensor data from the Aruba dataset in a smart home environment, showcases significant advancements in activity recognition with an F1 score of 0.758 using STA alone and an improved 0.863 when combined with an activity transition matrix. The study highlights STA’s efficiency and accuracy, with its minimal need for complex data preprocessing or extensive training, positioning it as a promising solution for unobtrusive elderly monitoring and anomaly detection in smart home buildings.Ghosh et al. [106] employed an online event-based activity discovery (OEAD) algorithm. Their method uses location and time features for clustering activity instances. The model achieved precision rates ranging from 0.40 to 1.00 and recall rates from 0.50 to 1.00 for various activities, demonstrating effective activity recognition. The algorithm particularly excelled in identifying infrequent activities critical for geriatric care, showcasing its potential for real-world applications in elderly monitoring systems.

With regard to detecting anomaly activity based on PIR sensors, Moshtaghi et al. propose statistical models for unobtrusively detecting abnormal periods of inactivity in older adults [114]. Eisa et al. introduced a system in their study to monitor the behavior of elderly individuals in their homes using PIR motion sensors [108]. This non-intrusive system aims to detect abnormal behaviors indicative of health or safety concerns. It processes sensor data to build a behavioral model, identifying deviations from normal patterns. In testing with synthetic datasets, the system showed high accuracy in recognizing various abnormal behaviors, such as oversleeping (96.26%) and less sleeping (97.71%) in the Profile A dataset, with slightly varying results in the Aruba dataset. Despite some challenges, such as lower accuracy in detecting ’NotBackHome’ behavior in the Aruba dataset (34.71%), the overall results demonstrate the system’s potential in supporting the independent living of elderly individuals by alerting caregivers to unusual activities that might signal health or safety issues. In the most recent effort to detect anomalous activities, Nazerfard et al. introduced a novel method that presents a ConvLSTM autoencoder (ConvLSTMAE) designed for identifying abnormal activities in elderly individuals [109]. This approach leverages ConvLSTM layers for encoding and decoding, effectively handling spatiotemporal data from smart home environments. The model’s performance was evaluated using the Aruba and Kyoto datasets, simulating behaviors such as sleep disturbances and confusion, common in dementia. ConvLSTMAE demonstrated superior performance in anomaly detection compared to traditional methods, showcasing its effectiveness in identifying abnormal behaviors without the need for specific labels during training. This marks a significant advancement in monitoring the health and safety of the elderly.

**Signal-based PIR sensors for activity detection:** In the context of signal-based activity detection using PIR sensors, we summarize signal-based approaches for activity detection with PIR sensors in Table 8. Luo et al. introduced an innovative indoor human activity recognition system in their work [115]. This system, designed for ambient assisted living (AAL) applications, effectively captures discriminative spatio-temporal features of human motion. The approach involves dividing the observed space into discrete sampling cells and employing Gaussian mixture model and hidden Markov model (GMM-HMM) for activity classification. The research specifically focuses on five activities: fall, sit down, stand up, walk, and run, achieving an average accuracy of 86.2%. In another innovative signal-based method utilizing PIR sensors for activity detection, Guan et al. introduce a system that effectively classifies six typical physical activities [116]. The system employs classification techniques such as the hidden Markov model and support vector machine, along with various machine learning techniques, including k-nearest neighbor, Gaussian mixture hidden Markov model (GM-HMM), naive Bayes, and SVM with different kernels. These methods are evaluated based on the time series and frequency spectrum of sensor signals, which serve as discriminative features for distinguishing activities. The approach leverages sparsity in IRC signals through compressive sensing, directly classifying sparse or compressible signals in the measurement domain. The system achieves high accuracy with a minimal number of sensors, showcasing its potential for smart home applications. The effectiveness in classifying human activities with over 97% accuracy using SVM with a linear kernel is particularly noteworthy, outperforming other machine learning methods.

In another study by Guan et al. [119], a state-of-the-art daily activity recognition system was developed. Central to the system’s effectiveness was the integration of a Gaussian mixture hidden Markov model (GM-HMM), a sophisticated machine learning technique adept at handling time-series data from PIR sensors. The GM-HMM, crucial for modeling sequences of activities, significantly enhanced the system’s accuracy by capturing the temporal dynamics and transitions between different states of activity. This machine learning core, skilled at interpreting complex sensor data, allowed for a detailed recognition of human activities, considering both spatial and temporal dimensions. Five types of activities were evaluated: normal walk, fast walk, siting down, squat-standing, and fall, with an average accuracy based on GM-HMM of about 91.6%. In another signal-based method for activity detection, Luo et al. developed a system that utilizes PIR sensors for indoor tracking and activity recognition [117]. It employs a network of sensor nodes with modulated fields of view (FOVs) and processes the PIR sensor signals for activity classification. Signal feature extraction utilizes short time energy (STE) due to the non-linear response of PIR sensors. A data fusion strategy is implemented to enhance localization accuracy. For activity recognition, a two-layer random forest classifier is employed, utilizing features like location, speed, and duration of activities. The system was tested in a mock apartment, achieving a mean localization error of about 0.85 m and over 92% accuracy in recognizing five types of daily activities (walking, sitting, lying, standing, and transitional). This innovative approach effectively combines spatial and temporal data for simultaneous tracking and activity analysis. In another analog-based activity detection research by Fujiwara et al. [48], an innovative activity recognition technique using an analog-output PIR sensor is developed, capable of identifying various activities at the same location. This technique leverages random forest machine learning, utilizing the frequency components of the sensor’s output as features. To optimize the feature set, a specific range of frequencies, defined by a starting frequency (SF) and an ending frequency (EF), was selected using a grid search method. The method’s efficacy was tested with five participants performing four different activities (eating, working with a PC, reading, and using a smartphone) while seated on a sofa. The results showed significant accuracy, with an F measure of 63.9% at an EF of 1.4 Hz, decreasing to 50% or lower when the SF exceeded 9.9 Hz. In this research, two PIR sensors were used—one on the ceiling and another in the corner—to cover people’s activities. Misaki et al. [118] proposed another system based on the random forest classifier, utilizing four PIR sensors to detect activity in different parts of the home. The average accuracy of the random forest classifier is over 90%. In their recent work [120], Liu et al. present an innovative approach to detecting human motion features using a PIR sensor array. The study employs a bidirectional long short-term memory (LSTM) neural network for recognizing various activities. The accuracy results for different actions are noteworthy: walking achieved 93%, jogging reached 97%, crouching down and squatting up both achieved high accuracies of 97% and 100%, respectively. Sitting demonstrated 65% accuracy, while standing up achieved 95%, and falling action reached a remarkable accuracy of 100%. This study showcases the effectiveness of their proposed method in accurately recognizing a diverse range of human activities based on PIR sensor data.

**Figure 14:**
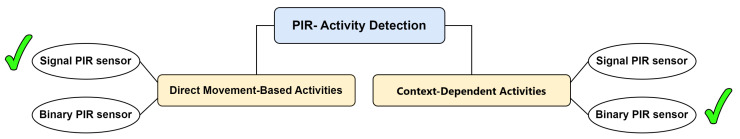
Activity detection based on PIR sensor.

Based on previous research, exploring activity detection through PIR sensors reveals two main approaches: direct movement-based activities and context-dependent activities. As you can see in Figure 14.

- **Direct Movement-Based Activities:**
  These activities are characterized by the direct physical movement they involve, which can be detected by a PIR sensor. Signal-based detection is considered superior to binary-based detection because it allows the reception of continuous signals. This continuous signal reception enhances the sensor’s ability to capture nuanced movements and variations in activity.
- **Context-Dependent Activities:**
  These activities are deduced based on the PIR sensor’s location and the assumption that movement in a specific area is associated with a particular activity. Previous research has shown that binary-based detection is widely used and effective for this context. The binary-based approach simplifies the analysis by focusing on the presence or absence of movement, making it popular and well-suited for inferring context-dependent activities.

## 4. Discussion

RQ1 concerned the predominant use of PIR sensor types, binary or signal-based, for capturing occupancy information. Based on the results, PIR sensors are widely used for capturing different levels of occupancy information (counting, localization, and activity detection) rather than mere detection. The popularity of PIR sensors primarily lies in localization, followed by activity detection, and lastly multi-person counting. With regard to the type of PIR sensors, the findings show that binary PIR sensors are more commonly used for activity detection, especially on a building scale. However, for localization purposes, signal-based PIR sensors are more popular. In terms of people counting, both signal-based and binary PIR sensors are equally utilized.

To answer RQ2, which pertained to the predominant machine learning algorithms or methods used for data processing based on PIR sensors, we considered the level of occupancy information and the type of PIR sensor data. For people counting, machine learning methods are not very popular with binary PIR sensors; however, they are more widely used for signal-based PIR sensors. Deep learning techniques, such as deep neural networks and convolutional neural networks, are more popular compared to algorithms that do not use deep learning. Among the non-deep learning algorithms, support vector machines are more prevalent. In localization tasks, the use of machine learning for binary-based data is not common. It is typically limited to tracking applications, where specific machine learning models are used, such as probabilistic neural networks and NBCL-naive Bayes classifiers. In contrast, for signal-based data, machine learning, especially deep learning algorithms, is quite popular. Deep learning-based methods surpass traditional machine learning algorithms in terms of popularity. The most commonly employed approaches involve a combination of convolutional neural networks, artificial neural networks, and long short-term memory networks. For methods that do not utilize machine learning in localization, the Kalman filter stands out as the most popular choice. Activity detection using PIR sensors significantly benefits from machine learning, making it a popular choice in this field. Most research in this area employs machine learning algorithms. In binary-based activity detection, both traditional machine learning and deep learning methods are popular. Random forest and support vector machines are commonly used for traditional approaches, while deep convolutional neural networks are more prevalent in deep learning-based methods, although algorithms like AdaBoost and recurrent neural networks also achieve good accuracy. Regarding signal-based activity detection, Gaussian mixture model hidden Markov models are more commonly used, but algorithms such as long short-term memory networks and random forest machine learning also yield good results. Additionally, based on the results, there is a direct link between the number of PIR sensors used and improved accuracy, but machine learning enhances accuracy with the same number of PIR sensors. For instance, in signal-based localization, greater accuracy is achieved when machine learning is utilized. Attaining the same level of accuracy without machine learning requires an increase in the number of sensors.

Regarding RQ3, the results indicate that the quantity and location of PIR sensors vary based on the designated level of occupancy information and sensor type. For people counting using binary sensors, placing sensors at the entrance is a more common practice. This typically involves placing one sensor outside and another inside, which facilitates counting the number of people. This setup is particularly effective for monitoring entrances, but it may not work accurately when multiple people enter simultaneously. This binary-based method is mostly used on a building scale, whereas signal-based methods for people counting are primarily used on a room scale. For the latter, placing four sensors, one in each corner of the room, is considered an effective approach. Overall, better accuracy is achieved when integrating both door-based and non-door-based methods. For localization, most studies consider the ceiling or the corners of walls as the best placement for sensors, regardless of sensor type. However, some studies have also explored using the floor for testing. In terms of the number of sensors required, binary sensors generally need a larger quantity to achieve the same accuracy as signal-based PIR sensors. For instance, most binary-based systems use more than 10 sensors, whereas for signal-based systems, a maximum of 4 sensors is typically sufficient in most studies. Additionally, the implementation of machine learning algorithms is often found to be more feasible with signal-based sensors. Therefore, signal-based PIR sensors are considered better for localization. Regarding the scale of use, binary sensors are predominantly utilized on a building scale, whereas signal-based sensors are more commonly used on a room scale. Regarding activity detection, binary PIR sensors are more common on a building scale. Most sensors used for this purpose are placed in doorways or near locations where a probable activity might occur, especially on walls. The number of sensors depends on the number of activities being monitored and the number of rooms. For signal-based systems, which are primarily used on a room scale, the ceiling and walls are the most common placement locations, particularly in areas directly in front of the person performing the activity.

**Figure 15:**
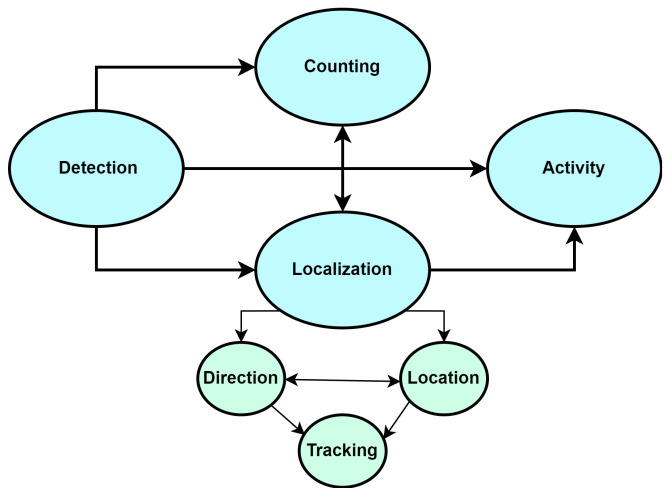
Occupancy information level connection based on PIR sensor.

In terms of RQ4, most binary PIR sensors used for detecting activities are implemented on a building scale, focusing on context-dependent activities. For instance, the movement of a person from one room to another is considered an activity. Binary sensors can be used to detect activities such as eating, sleeping, cooking, cleaning up, working or studying, going out, going to bed, and washing. Additionally, these sensors are employed for identifying travel patterns (direct, lapping, pacing, and random) and for detecting anomalies, including oversleeping, sleeping less, not being back home, being dead, or repeating actions. For signal-based sensors, most activity detection applications are on a small scale, concentrating on direct movement activities. The most significant activities detected by signal-based sensors include walking, sitting, lying down, standing, eating, working, falling, and doing anomalous activities. These detailed detections are due to the capability of signal-based sensors to capture more nuanced movements and variations in activity, as opposed to the binary PIR sensors, which mainly detect the presence or absence of motion. In the context of linking various levels of occupancy information using PIR sensors, the connections between these levels are illustrated in Figure 15. Detection is the foundational step, critical for subsequent processes like counting, activity recognition, and localization. Counting and localization are directly linked since understanding the number of people present can significantly enhance localization efforts. In addition, localization is closely connected to activity detection; namely, knowing an individual’s precise location within a space aids in identifying their specific activities. On the other hand, the relationship between counting and activity recognition is somewhat indirect, mediated through localization. The combination of knowing both the number of occupants and their locations provides a richer context for accurately interpreting activities. In terms of localization, particularly for tracing movements, it is crucial to have information about both the direction and the location of individuals. This dual knowledge enables a more precise tracking of movement patterns and paths within the monitored environment.

**Figure 16:**
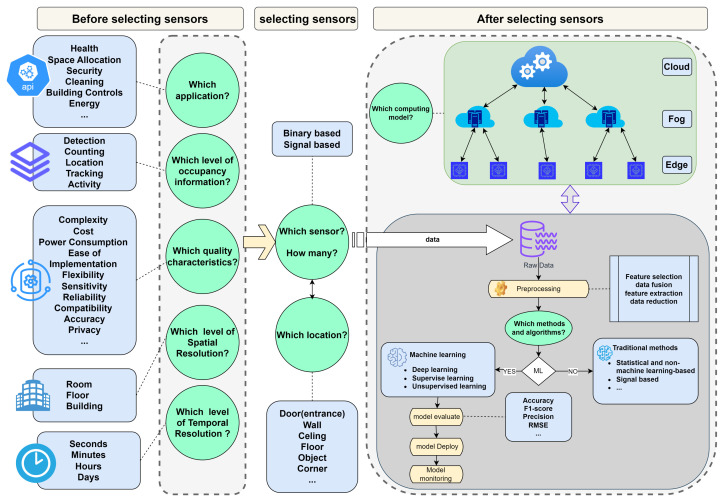
Decision-making framework for occupancy information in smart building based on PIR sensor.

To summarize our findings on the design choices for occupancy sensing systems, with a special focus on the decision-making framework for employing PIR sensors in smart buildings, the process is divided into two phases: before and after sensor selection, as you can see in Figure 16. In the pre-selection phase, attention is paid to the application’s domain, the level of occupancy information required, sensor quality characteristics, and the necessary spatial and temporal resolution. This comprehensive evaluation ensures the selection of the most suitable sensor type and its optimal placement to fulfill the established requirements. Following the selection of the sensor, attention shifts to data processing. Subsequently, the process involves choosing appropriate methods, which may encompass machine learning algorithms or non-machine learning approaches. In terms of machine learning, the performance of models is evaluated using metrics such as accuracy and RMSE. This leads to their deployment and continuous monitoring to guarantee effectiveness and adaptability to changing conditions. All this data-related processing can be executed using edge, fog, or cloud computing, offering flexibility in managing the collected information. In this study, we specifically focus on the level of occupancy information, sensor quality characteristics, spatial resolution, sensor location, and the methods employed, which include machine learning and other approaches. Future work could explore other aspects, such as application and further advancements in sensor technology.

## 5. Conclusions

The paper thoroughly examines the roles of binary and signal-based PIR sensors for occupancy information in smart buildings between 2015 and 2023. It highlights an increase in the number of articles across various levels of occupancy information, underscoring their unique and complementary functions. The findings show that signal-based PIR sensors are better at localization tasks, such as tracking movements and determining where humans are in a building or room, while binary PIR sensors are mostly used for activity detection across larger buildings, demonstrating their effectiveness in monitoring extensive areas. Moreover, these sensors can detect a range of activities, from simple movements like walking to more complex and anomalous behaviors, thereby providing valuable insights for security and behavioral analysis in smart buildings. Regarding people counting, both types of sensors are equally effective, underscoring their versatility in various environmental contexts. A significant aspect of the review is its focus on exploring the role of machine learning—particularly deep learning techniques such as deep neural networks and convolutional neural networks—in enhancing the functionality of PIR sensors. According to the findings, these advanced algorithms are particularly effective in processing signal-based PIR sensor data for people counting and localization, indicating a shift towards AI-driven approaches for improved accuracy and efficiency in occupancy sensing. The findings also emphasize the importance of strategic sensor placement and quantity. In particular, signal-based sensors achieve higher accuracy with fewer sensors, providing a more efficient approach to sensor deployment for precise occupancy detection. Moreover, this review highlights the link between different levels of occupancy information captured through PIR sensors. Overall, the review maps out the evolving role of PIR sensor technology in smart building management and underscores its crucial contribution to enhancing energy efficiency, security, and occupant comfort. It aims to further refine the capabilities of PIR sensors in smart building applications, thereby contributing to the development of more sophisticated and accurate building management systems. Accordingly, it sets the stage for future advancements in sensor technology and machine learning algorithms. We suggest conducting further research to explore the optimal non-invasive sensors, beyond PIR sensors, for capturing various levels of occupancy information, along with investigating methods to integrate these sensors with PIR sensors to develop a unified, integrated, or hybrid system. The investigation should leverage sensor fusion techniques, combining PIR sensors with various non-invasive sensor types, such as ultrasonic and environmental sensors. This strategy is expected to improve detection accuracy and broaden the range of activities. Additionally, exploring advancements in the sensitivity of PIR sensors, alongside comparisons with other types of infrared radiation-based sensors, is necessary. A critical examination of quality characteristics, including reliability, scalability and so on, in relation to the sensor type and the level of occupancy information is also vital. Furthermore, the role of edge computing and Edge AI should be examined for their potential to enhance real-time data processing and privacy.

## Figures and Tables

**Table 1:** Previous literature reviews on occupancy information.

Ref	Year	Main Focus	Occupancy Information Level	ML	Spatial Resolution	Consider PIR Sensors for All Levels of Occupancy Information
[33]	2012–2022	Focuses on the application area of occupancy information and sensor fusion based on a PIR sensor	Detection	Yes	Yes	No
[22]	1981–2021	Focuses on sensor fusion, especially for energy application	Detection and Prediction	Yes	Yes	No
[20]	2020	Compares different sensors for occupancy information	All	Yes	Yes	No
[30]	2018	Focuses on a privacy-preserved occupancy monitoring solution for people counting	Counting	No	Yes	No
[29]	2018	Compares different sensor types for the estimation and detection of occupancy	Counting and Detection	Yes	No	No
[34]	1998–2019	Examines the benefits and drawbacks of several occupancy detection techniques and provides a framework for comparison to help researchers choose the best sensors and algorithms	Detection	Yes	Yes	No
[21]	2009–2020	Focuses on occupancy detection for HVAC based on different types of buildings	Detection	Yes	Yes	No
[35]	2009–2021	Provides an overview of environmental sensors used for occupancy detection/estimation; proposes a technique to calculate	Detection, Counting	Yes	Yes	No
[36]	2012–2021	Focuses on application (which sensor is good for which application)	Detection, Counting, Identity, Tracking	No	Yes	No
[24]	2009–2020	Explores forecasting algorithms for occupancy information	Counting, Detection, Location	Yes	Yes	No
[37]	2012–2022	Conducts human–building interaction research	Counting, Detection, Location, and Tracking	No	Yes	No
[32]	2011–2021	Provides a selection of occupancy measurement systems for different ranges of people and the occupancy counting accuracy situation of different measurement systems and algorithms	Counting	Yes	No	No
[26]	2020	Reviews occupancy measurement systems based on different sensors, especially image/video-based methods; analyzes and discusses their applicable scopes and limitations	Detection, Counting, Identity, Track, Location	Yes	No	No
[25]	2023	Explores sensing within smart buildings	All	No	Yes	No
[31]	2015–2022	Examines how deep learning and transfer learning methods are used for occupancy detection	Detection	Yes	Yes	No
[38]	2020	Presents a thorough analysis of device-free developments in indoor localization and tracking in multi-resident environments	Location, Tracking	Yes	Yes	No
Our Research	2015–2023	Reviews methodologies and machine learning approaches for occupancy information based on PIR sensors	Detection, Counting, Location, Tracking, and Activity	Yes	Yes	Yes

**Table 2:** Search String.

(“passive infrared sensor” OR “PIR sensor” OR “infrared sensor”)AND(“occupaäncy” OR “occupant” OR “people estimate*” OR “people activity*” OR “human activity*” OR “people monitor*” OR “people count*” OR “people track*” OR “people movement” OR “people position” OR “people location” OR “people speed” OR “activity detect*” OR “activity recognition*” OR “Movement detection” OR “movement recognition” OR “Tracking detection” OR “tracking recognition” OR “Position detection” OR “Location detection”OR “trajectory detection” OR “Target detection” OR “human count*” OR “Action recognition” OR “indoor activity” OR “building management*” OR “building monitoring*” OR “speed detection*” OR “speed recognition*”)

**Table 3:** Inclusion and Exclusion Criteria.

Inclusion Criteria	Exclusion Criteria
Articles focusing on only PIR sensors, or where PIR sensors are the primary sensor for people counting, location tracking, and activity monitoring.	Articles are primarily concerned with hardware development or enhancing the sensitivity of PIR sensors.
Articles are written in English.	Thesis, books, and preprint studies.
Research on occupancy information where PIR sensors play a main sensors.	Studies involving sensor fusion where PIR sensors are not the main sensor.
-	Duplicate publication.
-	Another type of infrared-based sensor.

**Table 4:** Comparison of Binary-Based and Signal-Based PIR Sensors.

Aspect	Binary-Based PIR Sensors	Signal-Based PIR Sensors
Output Type	Binary (On/Off)	Analog signal that varies with infrared intensity
Complexity	Simple	Complex
Data Provided	Presence or absence of motion	Detailed information like size, speed, and direction of the object
Cost	Generally lower	Higher due to more complex processing requirements
Power Consumption	Lower	Higher due to continuous signal processing
Ease of Implementation	Easier to implement and integrate	Requires more sophisticated setup and calibration
Flexibility	Less flexible in terms of information gathering	Highly flexible in terms of the range of information
Sensitivity	Less sensitive to minor variations	Highly sensitive to even slight variations
Reliability	Reliable for basic people counting and presence detection; may not differentiate well between complex activities or provide precise localization.	Highly reliable for localization and detailed occupancy analysis, including people counting, due to the ability to detect direction and speed of movement.
Compatibility	Compatible with simple control systems	May require integration with more advanced systems or data processing units

**Table 7:** Binary PIR Sensors for activity Detection.

Ref	Output	Location	Spatial Resolution	Type of Activity	Algorithms
[95]	Binary	11 PIR (wall), 8 (Door)	Buildings	Eat, Bathroom activity, Sleep, Cook, Clean-up, Living room activity, Work and Study with PC, Go out	Accuracy, Random Forest (62.8%)
[96]	Binary	(2 modules each 5) Ceiling	Buildings	Going to bed, Going to the coffee table, Eating, Going to the bathroom, Entering the room, Exiting the Room,	Random Forest, Extremely Randomized Trees, Support Vector Machines (SVM), Naive Bayes, Ada Boost, Logistic regression, and kNN
[97]	Binary	27 (different parts) (4 door sensors)	Buildings	Bed to Toilet, Eating, Meal Preparation, Relax	Average accuracy, DCNN classifier (99.36%)
[98]	Binary	31 (different places) (4 door)	Buildings	Bed to Toilet, Eating, Enter Home, Housekeeping, Leave Home, Meal Preparation, Relax, Sleeping, Work, Wash Dishes	Accuracy, DCNN (99.23%)
[99]	Binary	25 (different places) (4 door)	Buildings	Bed to Toilet, Eating, Enter Home, Housekeeping, Leave Home, Meal Preparation, Relax, Sleeping, Work, Wash Dishes	F1-score, Scanpath Trend Analysis (STA) (86%)
[100]	Binary	27 (different parts) (4 door sensors)	Buildings	Travel Patterns (Direct, Lapping, Pacing, Random)	Accuracy (Naive Bayes (82.51%), one vs. rest (90.46%), KNN (93.25%), Decision Tree (93.58%), SVC (93.81%), Gradient Boost (94.06%), Random Forest (94.48%), DCNN (97.84%))
[101]	Binary	27 (different parts) (4 door sensors)	Buildings	Bed to Toilet, Eating, Enter Home, Housekeeping, Leave Home, Meal Preparation, Relax, Sleeping, Work, Wash Dishes	Accuracy, RNN (98.148%)
[102]	Binary	27 (different parts) (4 door sensors)	Buildings	Get House, Leave House, Take Food, Bed to Washroom, Rest, Food Preparation, Sleep, Work, House Cleaning, Washing Utensils, Simple Exercise, Pick Objects	Accuracy, DCNN (98.68%)
[103]	Binary	31 (different places) (4 door)	Buildings	Meal Preparation, Relax, Eating, Work, Sleeping, Wash Dishes, Bed to Toilet, Enter Home, Leave Home, Housekeeping	Accuracy, AdaBoost (98%)
[104]	Binary	16 (wall)	Room	Heavy, medium, light, and resting activities	Accuracy, SVM (99.7%)
[105]	Binary	31 (different places) (4 door)	Buildings	Bed to Toilet, Eating, Enter Home, Housekeeping, Leave Home, Meal Preparation, Relax, Sleeping, Work, Wash Dishes	F1 scores, DeepLSTM (90.3%), Deep 2D-CNN (90.8%), Deep 2D-CNN_*L*_*STM* (91.9%)
[106]	Binary	31 (different places) (4 door)	Building	Sleeping, Bed to Toilet, Meal Preparation, Relax, Eating, Wash Dishes, Work	Average Precision, Online Event-Based Activity Discovery (87%)
[107]	Binary	31 (different place) (4 door)	Buildings	10 Activities	Accuracy, DCR-OL 82.23%
[108]	Binary	31 (different place)	Building	OverSleeping, LessSleeping, NotBackHome, Dead	Accuracy, Rule-based Anomaly Classification (72.93%)
[109]	Binary	31 (different place) (4 door)	Buildings	Repetition, Disturbance in sleep, and Confusion	Accuracy, ConvLSTM AE (90.3%), LSTM AE (89%), LSTM (81%), OCSVM (70%)
[110]	Binary	31 (different place) (4 door)	Buildings	Meal Preparation, Relax, Eating, Work, Sleeping, Wash Dishes, Bed to Toilet, Enter Home, Leave Home, Housekeeping	Accuracy, marker-based stigmergy and a Directed-weighted Network (DwN) (96.69%)
[111]	Binary	4 Wall	Buildings	Washing Dish, Resting, Eating, Resting while Eating, Cooking	Accuracy, PCA-KNN (94%)

**Table 8:** Signal-based activity detection.

Ref	Output	Location	Spatial Resolution	Type of Activity	Algorithms
[117]	Signal	9 Ceiling (near together)	Room	Walking, Sitting, Lying, Standing, Transitional	Accuracy, First layer RF (0.82), Second layer RF (0.93), SVM (0.79), Naive Bayes (0.66)
[48]	Signal	2 (Ceiling, Corner)	Room	Eat, Working with PC, Reading, Smartphone	Precision, Random Forest (64.7%)
[118]	Signal	4 (Wall), 1 (Ceiling) in each room	Buildings	Bath Room (absence, Stationary, toothbrushing, using laundry, washing hands), Bed Room (absence, changing clothes, sleeping, stationary), Living room (absence, eating, reading, stationary, writing), Kitchen (absence, cutting, frying, stationary, using fridge)	Average accuracy, Random Forest (over 90%)
[115]	Signal	1 (Ceiling)	One room	Fall, Sit down, Stand up, Walk, Run	Average accuracy, GMM-HMM (86.2%)
[116]	Signal	(3 modules each 4), 1 Ceiling, 2 Sides of person	Room	Walk, Run, walk with payload, Walk while waving right hand, Walk with a squat-standing, Fall	Accuracy, KNN (74.38%), GM-HMM (96.67%), Naive Bayes (85.42%), SVM (Linear kernel) (97.71%), SVM (multinomial kernel) (96%), SVM (RBF) (85.63%)
[119]	Signal	(4 modules each 4) one ceiling, 3 sides of person	Room	Normal walk, fast walk, sit down, squat-standing, fall	Accuracy, GM-HMMs (91.6%)
[120]	Signal	5 (bar near wall)	Room	Walking (93%), Jogging (97%), Crouching down (97%), Squatting up (100%), Sitting (65%), Standing Up (95%), Falling action (100%)	Accuracy, LSTM (92.42%)
[40]	Signal	4 Ceiling	Room	Anomaly activity	Accuracy, LC-MCAS-DL (93.55%)

## Data Availability

Data are contained within the article.
